# The impact of multiple agricultural land uses in sustaining earthworm communities in agroecosystems - A global meta-analysis

**DOI:** 10.1038/s41598-024-81676-5

**Published:** 2024-12-04

**Authors:** Bibiana Betancur-Corredor, Andrey Zaitsev, David J. Russell

**Affiliations:** 1 Center for Development Research (ZEF) - University of Bonn, Genscherallee 3, 53113 Bonn, Germany; 2 Senckenberg Museum für Naturkunde Görlitz, Görlitz, Germany; 3 Bonares Center for Soil Research, Halle, Germany

**Keywords:** Ecology, Biodiversity

## Abstract

The impact of agricultural land use on biodiversity has been extensively examined through efforts to synthesize available data. Nevertheless, there is still a lack of a thorough synthesis describing the earthworm response to agricultural land-use Our meta-analysis compared undisturbed ecosystems (i.e., undisturbed grassland, primary forest) as controls against agricultural land-use treatments, with data extracted from 113 publications across 44 countries, yielding 1040 pairwise comparisons of earthworm density and biomass, and 536 pairwise comparisons of earthworm diversity. We also evaluated how agricultural land use effects on earthworms depend on soil, climate, and management practices reported in the studies. Arable cropland had significantly lower earthworm density (-18%), biomass (-15%), and species richness (-27%) compared to undisturbed sites. Conversely, higher earthworm density, biomass and species richness were observed in pastures, sites under agroforestry, crop management with fallow periods and crop-livestock integration. In regions with continental climates characterized by cool summers, agricultural land use exhibited positive effects on earthworm density and biomass. In sites with higher soil bulk density and lower organic matter content the adverse effects of agricultural land use are exacerbated. However, highly heterogeneous earthworm responses cannot be explained by the individual evaluation of climatic, soil-related, or management factors. Our results guide future efforts to address the data limitations that enable us to enhance our understanding of the interactive effects of various factors on earthworm responses to agricultural land use, as well as evidence-based and management strategies targeted at promoting sustainable agricultural systems while preserving soil biodiversity.

## Introduction

Earthworms are keystone fauna in terrestrial ecosystems, playing crucial roles in soil health and ecosystem functioning^1^. Earthworms play a crucial role in enhancing nitrogen mineralization^2^, and contribute to improving soil structure by enhancing soil macroaggregates and macropores through their burrowing and casting activities^3^. Agricultural land use refers to the deliberate activities taken to enhance the productivity or profitability per unit area of rural land use, including both targeted practices within an agricultural land use and the transition between land uses. Agricultural land use can pose significant threats to earthworm populations worldwide in terms of density and diversity^4^. The shift from extensively to intensively managed agroecosystems is often seen as one of the main drivers of biodiversity decline in global agroecosystems^5^, and is considered the main factor applying pressure on soil biodiversity^6^. Shifting from extensive to intensive management can also generate a feedback loop, as high input systems can enhance productivity while modifying the soil faunal community such that artificial inputs are constantly required to sustain productivity^7^.

Some of the agricultural land uses increase the agrochemical use to enhance crop yields and control pests, diseases, and weeds^8^. For example, the conventional agricultural land use refer to the adoption of mechanized farming practices and irrigation systems which streamline agricultural operations, reduce labor requirements, and increase overall productivity^9^. Monoculture, the practice of cultivating a single crop species over large areas, is a common strategy in conventional agricultural land use. Simplified crop systems focus on a limited number of high-yielding crop varieties, allowing for standardized management practices and efficient resource allocation^10^. Conventional agriculture also often relies on intensive tillage practices to prepare soil for planting, control weeds, incorporate crop residues and organic matter inputs. While tillage can improve seedbed preparation and short-term nutrient availability, it can also lead to soil erosion, compaction, and degradation over time^11^.

One alternative approach to increasing agricultural production in a more sustainable manner is the use of natural processes to substitute agrochemicals, while still preserving or boosting yield^12^. Examples include developing field edges to support natural enemies of crop pests, to reduce crop damage, and to reduce the use of pesticides, or cover-cropping or intercropping with legumes to populate fields with nitrogen-fixing bacteria that improve soil fertility and increase crop yields^13^. Rather than relying on external inputs, agroforestry or conservation agriculture are low-input and resource-conserving systems that support favorable ecological interactions within the agroecosystem^14^. Although there is no single specific management technique or system that is referred to as sustainable agriculture^15^, it can include a combination of small-scale/patch farming, integrated pest management, conservation agriculture, pasture and forage systems, tree incorporation, irrigation management, and the incorporation of agricultural biodiversity into farming systems^16^.

The effect of varying levels of intensity of individual agricultural practices on earthworms have been explored via global meta-analyses. Systematically analyzed data from over 70 long-term field experiments^17^showed that the relative effects of the adoption of less intensive practices (organic agriculture, no-tillage) on earthworm communities is dependent on soil properties. A meta-analysis (165 publications performed over 65 years) showed that less intensive soil cultivation practices (e.g. no-tillage and conservation agriculture) lead to higher earthworm abundance and biomass (especially of epigeic and anecic species groups)^18^, with higher responses of earthworm density in fine-textured soils in warm temperate zones. A meta-analysis also emphasized a trend of increased earthworm abundance and species richness following transitions to lower intensity land uses, although these authors highlight that changes in soil properties that may occur during conversion can result in positive or negative effects on community size and diversity^19^.

With this respect, meta-analysis offers a powerful tool to synthesize data from multiple studies, providing a comprehensive understanding of the collective effects of agricultural land use on earthworm communities. In this article, we present a meta-analytic approach to examine the influence of different forms of agricultural land use on earthworm populations across the globe. While earlier meta-analyses^19–21^ have demonstrated the effects of agricultural land use on biodiversity, they have not differentiated the specific effects on earthworms. Specifically, we investigate the effects of various agricultural land uses on earthworm density, diversity and biomass. For this purpose, we conducted a meta-analysis in which the control group consisted of undisturbed ecosystems reported by the studies, such as undisturbed grasslands, primary forests, and secondary forests with more than 30 years of establishment. These undisturbed sites served as a reference point for comparison with the treatment group, which represented various forms of agricultural land use This approach allows us to systematically evaluate the effects of different agricultural land uses relative to ecosystems that have remained largely unaffected by human intervention. We explore the role of climatic conditions and soil properties in shaping earthworm responses to agricultural land use. Furthermore, we assess publication bias and heterogeneity in the literature to ensure the robustness and reliability of our meta-analytic findings.

## Results

### Literature search

**Fig. 1:**
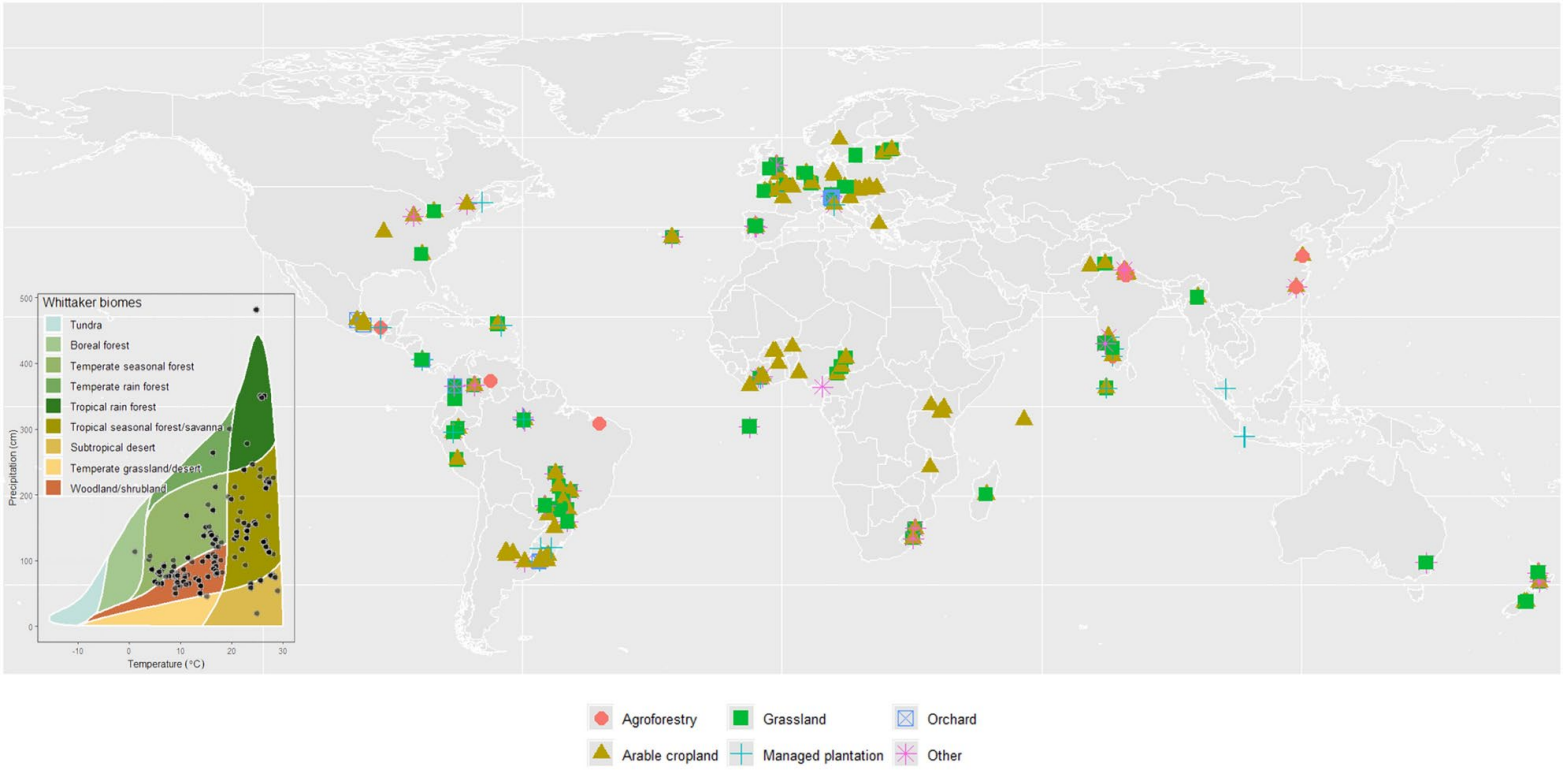
Global distribution of study sites indicating the agricultural land use studied at each research site. Colors and symbols represent agricultural land use types. The left inset represents the distribution of study sites within main biomes.

A total of 11338 references were retrieved using the refined search strategy (Supplement 1). The initial screening of the studies based on title and abstract reduced the number of relevant references to 134 studies assessing the effects of agricultural land use on earthworms. After examining their full text, 113 publications fulfilled all inclusion criteria and reported the necessary information for meta-analysis inclusion. The full list of studies can be found in Supplement 2. The final dataset included publications across 44 countries in 146 locations (Fig. 1), yielding 1040 pairwise comparisons of earthworm density and biomass, and 536 pairwise comparisons of earthworm diversity. In terms of forms of agricultural land use, 13 different treatments were included, four different cropping systems and different concurrent management practices such as tillage, pesticide and fertilizer application (for more details on each treatment, please refer to the Methods: Search strategy section). The studies are predominantly located in South America (35 publications), Europe (29 publications) and Asia (12 publications).

### Effects of agricultural land-use

In order to examine the true effects of agricultural land use on a given site under identical climatic and soil conditions - where the only difference is in agriculture management - undisturbed areas were used as reference for the estimation of the effects on density, biomass and diversity indices. If our meta-analysis showed no significant effects for a particular factor, it is not always unfavorable. Overall, there were varied and wide-ranging effects on biomass and earthworm density. The effects on earthworm density were estimated based on the analysis of 691 pairwise comparisons, resulting in response ratios between −18.8059 and 17.7513, with 50% of the estimations being negative. To estimate the effects on earthworm biomass, the analysis comprised 283 pairwise comparisons, and response ratios ranged from −14.0079 to 16.0344, with 52% of the estimations being positive.

**Fig. 2:**
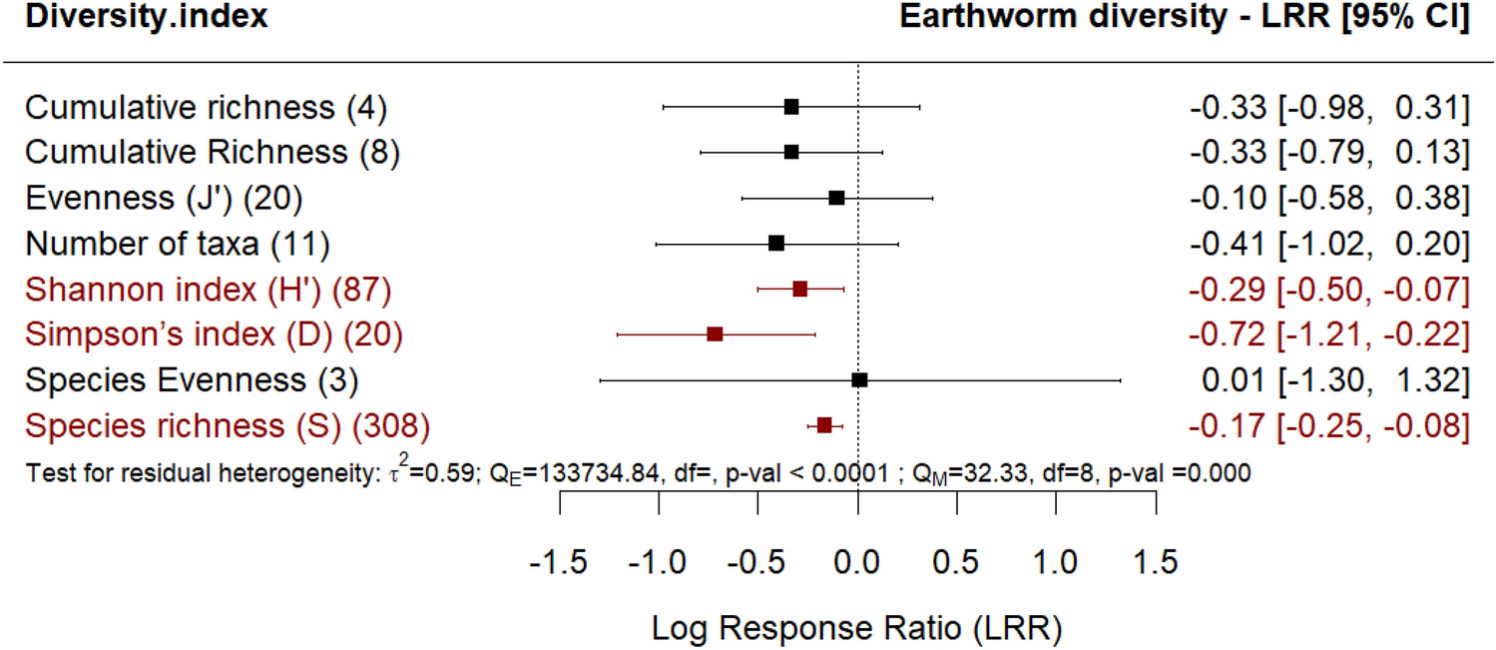
Effect of agricultural land use on earthworm diversity, estimated for the various diversity indices documented in the studies in comparison with undisturbed sites (e.g., primary forest, undisturbed grasslands). The following forest plot illustrates the average effect (squares), with the 95% confidence intervals presented in brackets, and the sample sizes (number of pairwise comparisons) for each group enclosed in parentheses.Significantly negative effects are colored in red. Significantly positive effects are colored in green.

Arable cropland and managed plantations compared to undisturbed sites had less diverse earthworm communities, with significant results reported as Shannon Index (H’), Simpson’s index (D) and species richness (S) (Fig. 2). These outcomes reflect the negative impact of agricultural land use on earthworm communities, including loss of species richness, decreased evenness in species abundances, and potential dominance of a few resilient species. Since earthworm species richness was the diversity metric most commonly reported, it was selected for use in the following meta-regressions and moderator analyses.

**Fig. 3:**
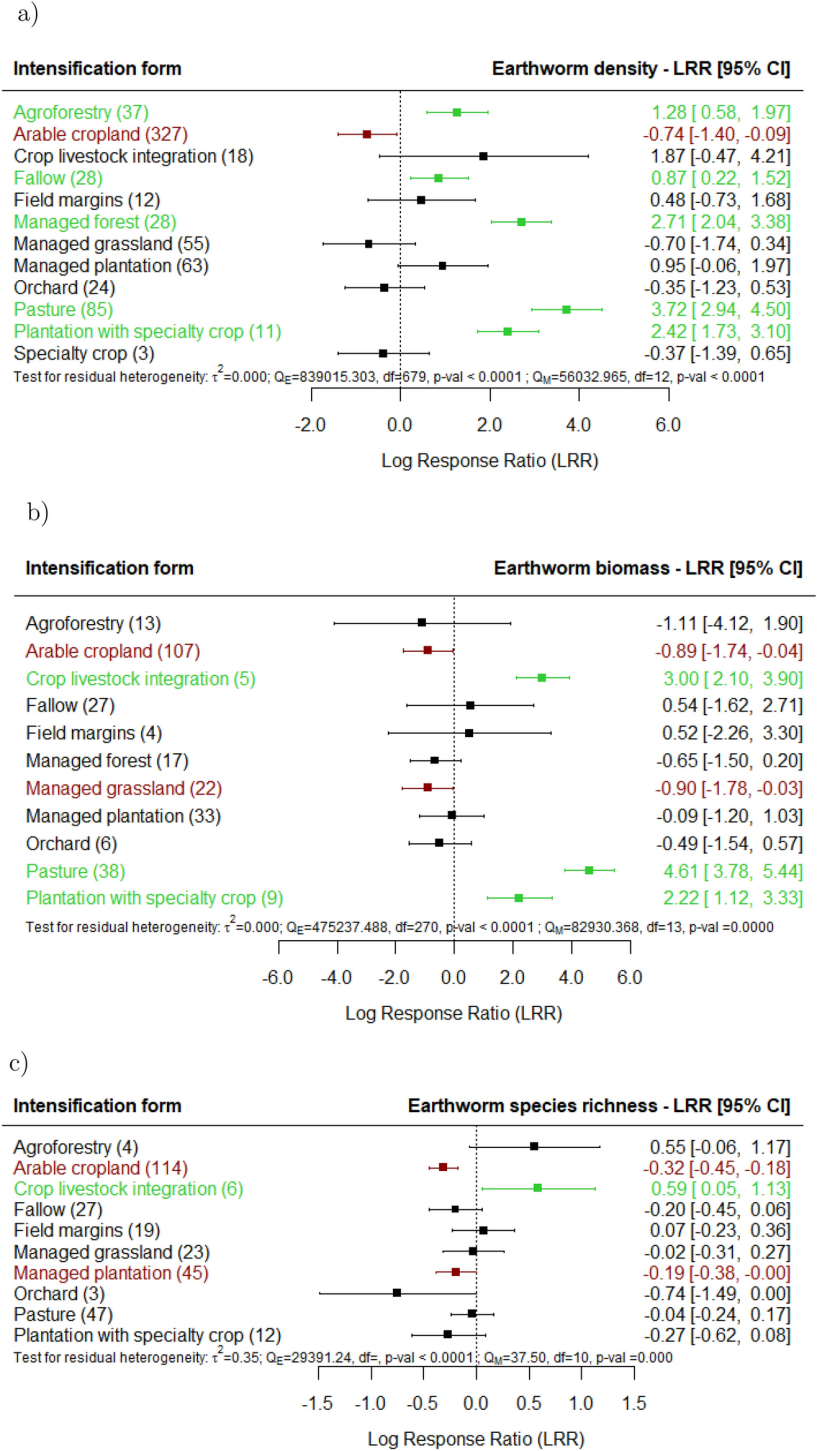
Effect of agricultural land use on earthworm (**a**) density, (**b**) biomass and (**c**) species richness, for the various land uses documented in the studies in comparison with undisturbed sites (e.g., primary forest, undisturbed grasslands). The following forest plot illustrates the average effect (squares), with the 95% confidence intervals presented in brackets, and the sample sizes (number of pairwise comparisons) for each group enclosed in parentheses.Significantly negative effects are colored in red.

Our findings reveal that arable croplands have lower earthworm density, biomass, and species richness compared to undisturbed sites. Conversely, positive effects on earthworm density were observed in pastures, agroforestry, and crop management with fallow periods (Fig. 3a). Moreover, pastures and sites under crop-livestock integration showed significantly higher earthworm biomass (Fig. 3b), while only crop-livestock integration showed higher species richness (Fig. 3c). However, managed plantations had significantly lower species richness. Agricultural land uses employing field margins or orchards do not have a significant effect on earthworms.

**Fig. 4:**
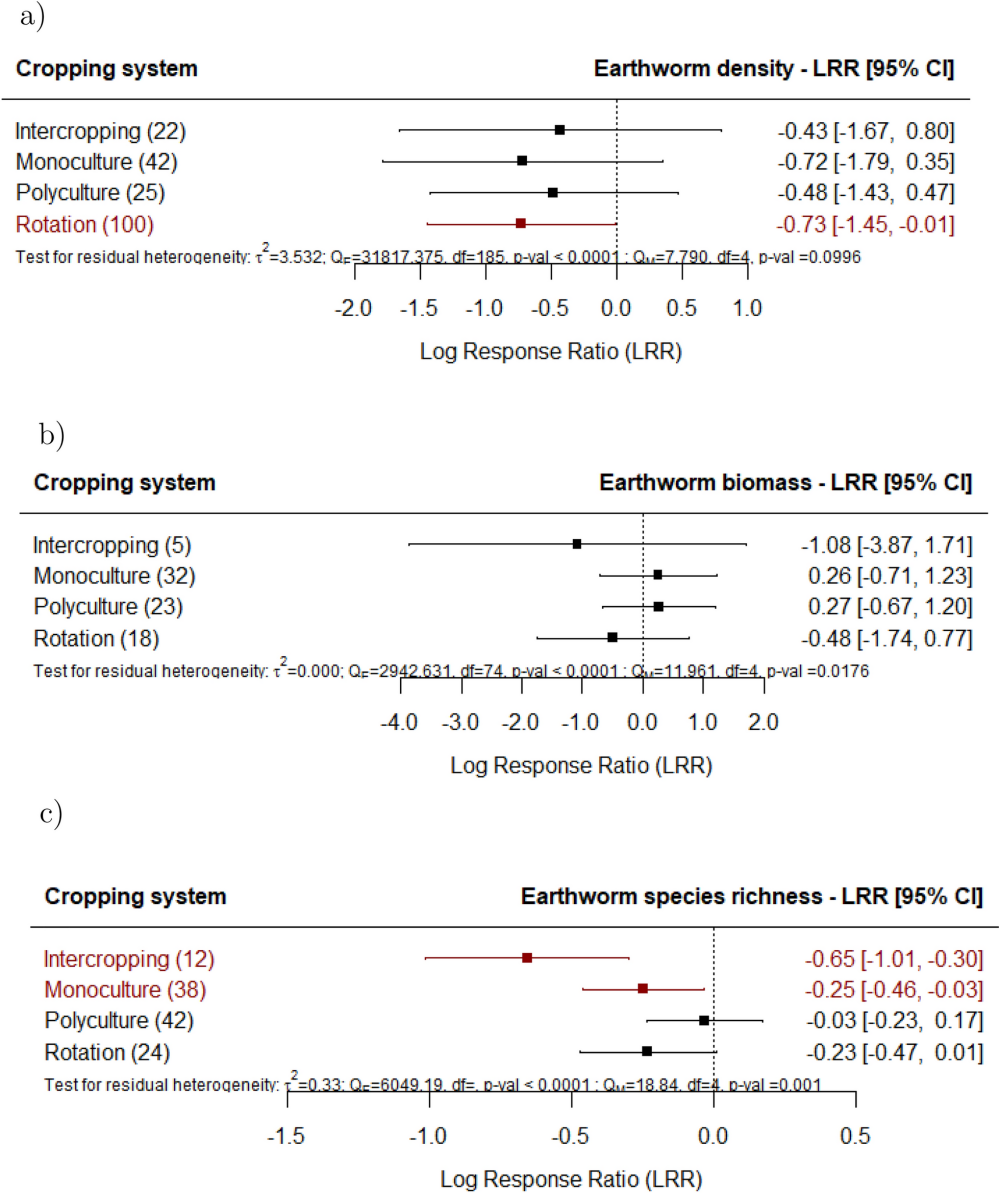
Effect of agricultural land use on earthworm (**a**) density, (**b**) biomass and (**c**) species richness, for the various cropping systems documented in the studies in comparison with undisturbed sites (e.g., primary forest, undisturbed grasslands). The following forest plot illustrates the average effect (squares), with the 95% confidence intervals presented in brackets, and the sample sizes (number of pairwise comparisons) for each group enclosed in parentheses. Significantly negative effects are colored in red.

To gain a deeper understanding of the negative effects observed in arable croplands, we examined the effects of various cropping systems (Fig. 4a-c) and found that intensive rotations (i.e., cereal-legume) have significantly lower earthworm density and diversity reported as Shannon-Index (Supplement 3), while intercropping and monoculture have lower species richness compared to undisturbed sites. Intercropping and polyculture do not have significantly different earthworm density and biomass. Further meta-regressions were conducted to examine the impact of agricultural land use on earthworms depending on the duration of land use. The results showed that the effects of agricultural land use were not significantly influenced by the duration of land use, meaning that the short or long duration of land use did not significantly impact the outcomes reported in the studies (Supplement 4).

**Fig. 5:**
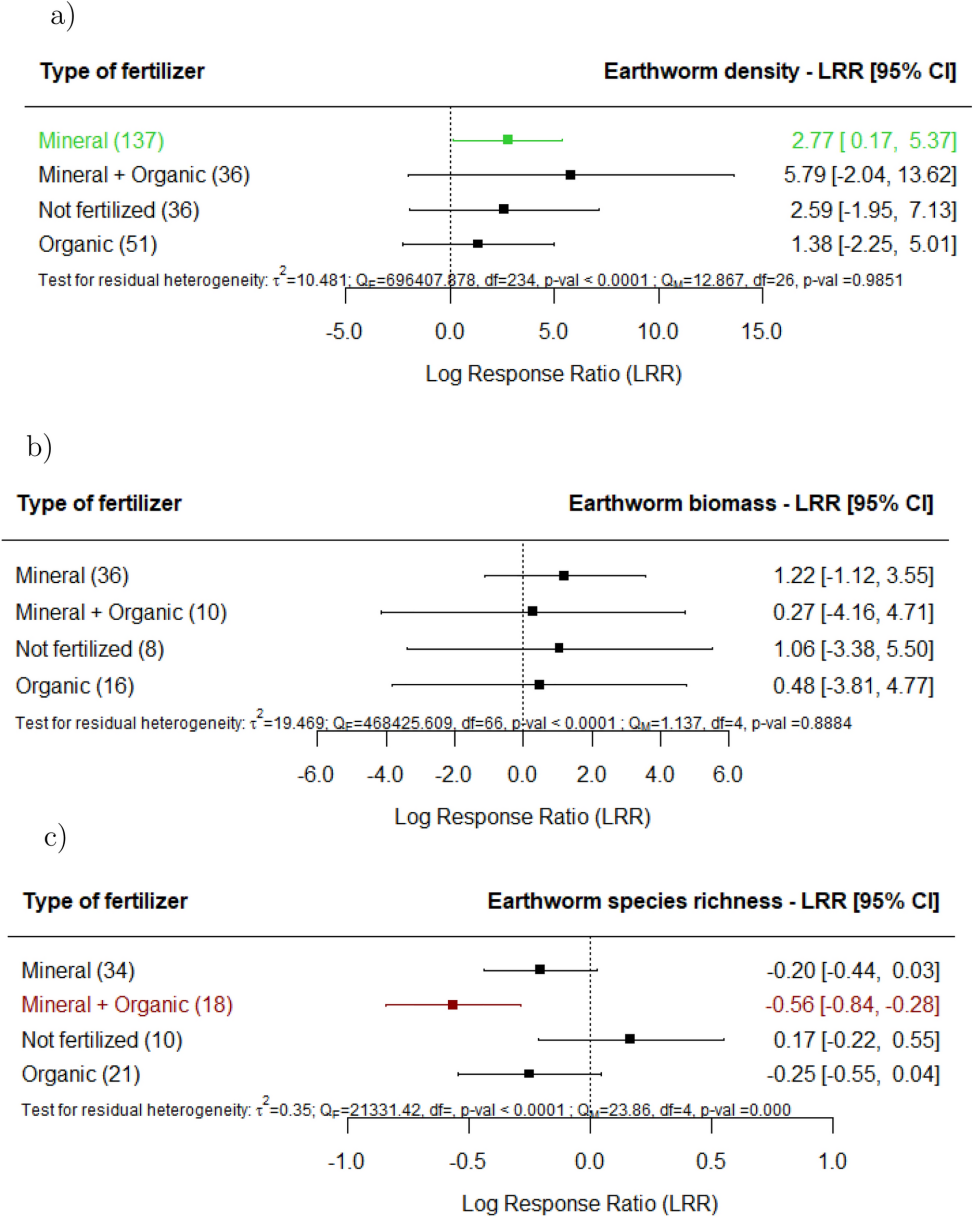
Effect of agricultural land use on earthworm (**a**) density, (**b**) biomass and (**c**) species richness, for the various types of fertilizer documented in the studies in comparison with undisturbed sites (e.g., primary forest, undisturbed grasslands). The following forest plot illustrates the average effect (squares), with the 95% confidence intervals presented in brackets, and the sample sizes (number of pairwise comparisons) for each group enclosed in parentheses. Significantly negative effects are colored in red. Significantly positive effects are colored in green.

**Fig. 6:**
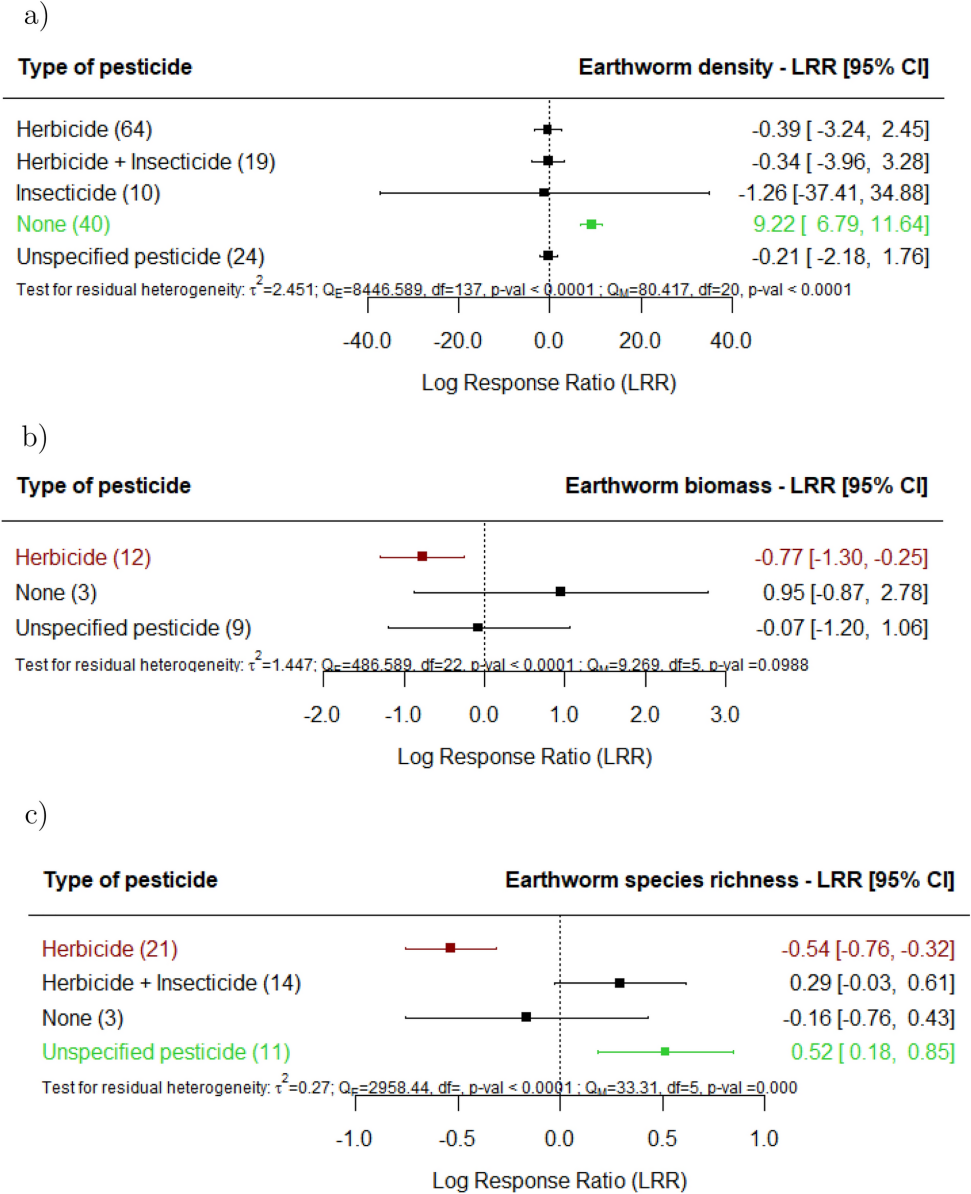
Effect of agricultural land use on earthworm (**a**) density, (**b**) biomass and (**c**) species richness, for the various types of pesticide documented in the studies in comparison with undisturbed sites (e.g., primary forest, undisturbed grasslands). The following forest plot illustrates the average effect (squares), with the 95% confidence intervals presented in brackets, and the sample sizes (number of pairwise comparisons) for each group enclosed in parentheses.Significantly negative effects are colored in red. Significantly positive effects are colored in green.

**Fig. 7:**
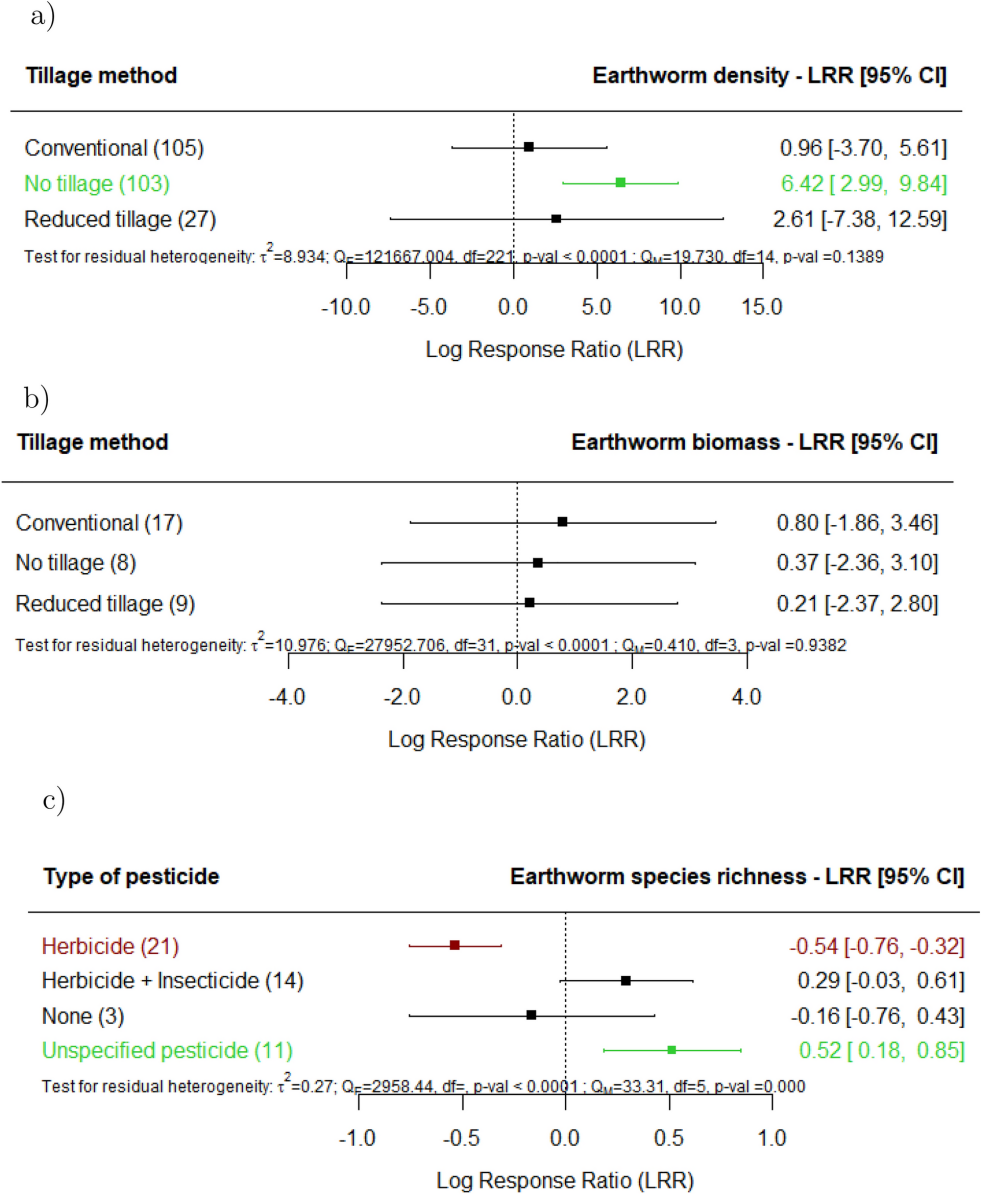
Effect of agricultural land use on earthworm (**a**) density, (**b**) biomass and (**c**) species richness, for the various tillage methods documented in the studies in comparison with undisturbed sites (e.g., primary forest, undisturbed grasslands). The following forest plot illustrates the average effect (squares), with the 95% confidence intervals presented in brackets, and the sample sizes (number of pairwise comparisons) for each group enclosed in parentheses.Significantly negative effects are colored in red. Significantly positive effects are colored in green.

Regarding the effects of specific management techniques, when mineral fertilizers are applied (Fig. 5a-c), significant increases on earthworm density are observed; nevertheless, when mineral is combined with organic fertilization, we detected significantly lower earthworm species richness and Shannon-Index (Supplement 3). When organic fertilizers are applied, we did not observe significant effects on earthworms. It is noteworthy that earthworm density significantly increased in fields where agricultural land use did not involve pesticide treatment (Fig. 6a). Herbicide spraying resulted in a decrease in the biomass (Fig. 6b) and species richness (Fig. 6c) of earthworms. Earthworm density increased in fields where agricultural land use utilized no tillage techniques (Fig. 7a), but biomass changes were not explained by tillage intensity (Fig. 7b). There was a detrimental effect on species richness in fields where agricultural land use utilized reduced tillage (Fig. 7c). No significant effects were found on earthworm density or biomass in fields where agricultural land use utilized conventional or reduced tillage.

### Climatic factors

**Fig. 8:**
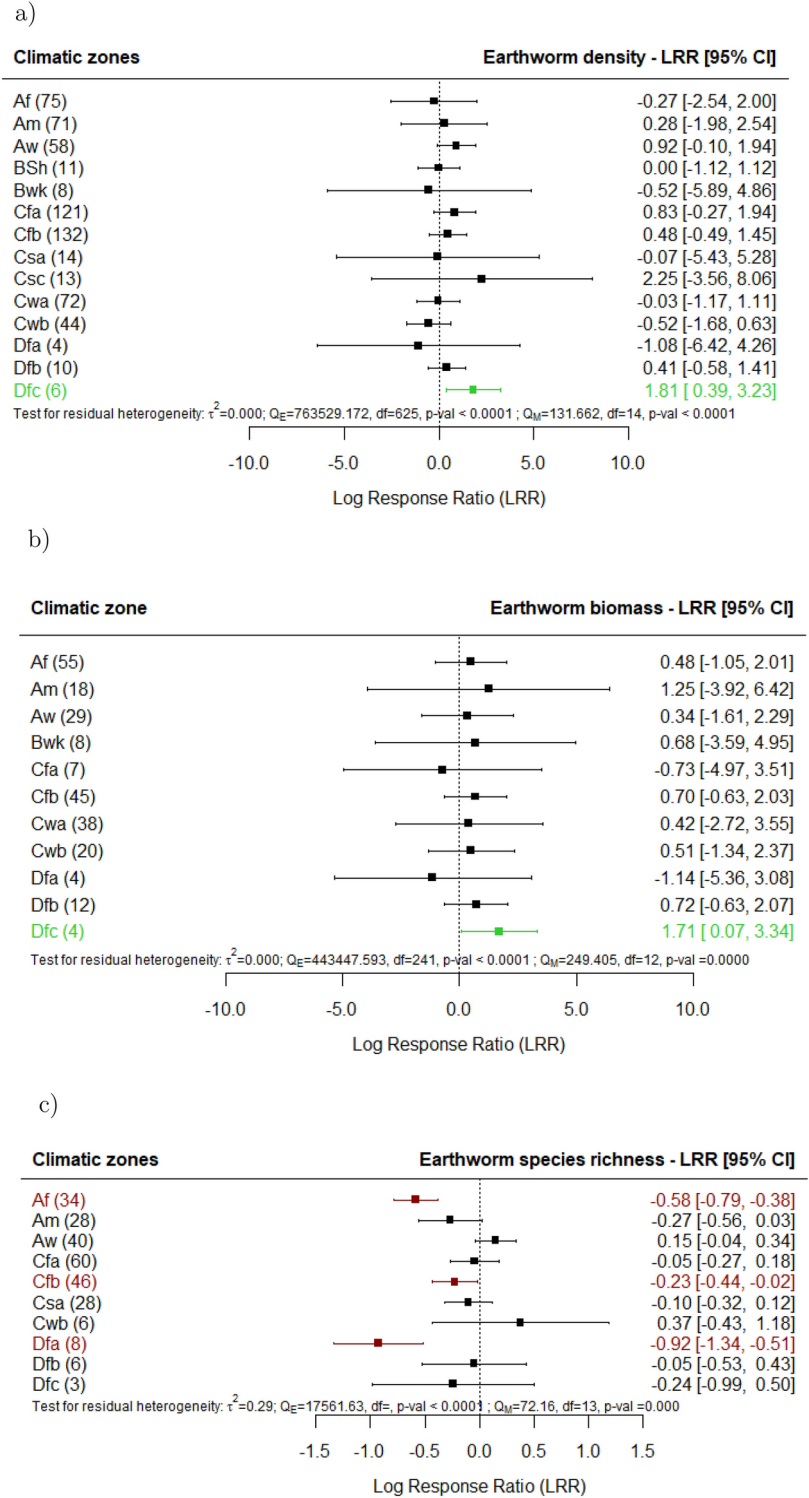
Effect of agricultural land use on earthworm (**a**) density, (**b**) biomass and (**c**) species richness, for the climatic zones corresponding to each research site documented in the studies in comparison with undisturbed sites (e.g., primary forest, undisturbed grasslands). The following forest plot illustrates the average effect (squares), with the 95% confidence intervals presented in brackets, and the sample sizes (number of pairwise comparisons) for each group enclosed in parentheses. Significantly negative effects are colored in red. Significantly positive effects are colored in green. The scheme symbols are described as: A (Tropical), B (Dry), C (Temperate) and D (Continental); f (Rainforest; no dry season), m (Monsoon), w (Dry winter), s (Dry summer), S (Semi-Arid Steppe, h (Hot) k (Cold), a (Hot summer), b (Warm summer), c (Cold summer).

The synthesized studies showed that when agricultural land use was carried out in locations with a continental climate, no dry season, and cool summers (Dfc), agricultural land uses had higher earthworm density (Fig. 8a) and biomass (Fig. 8b) compared to undisturbed sites. Sites with tropical rainforest climates (Af), temperate climates (Cfb), and continental climates (Dfa) with no dry season and hot summers showed lower species richness (Fig. 8c) and Shannon Index (Supplement 3) in agricultural land use in comparison with undisturbed sites. No significant correlation was found between the mean annual precipitation reported in the research sites and the agricultural land use effects on earthworm density, biomass, and species richness (Supplement 4).

### Soil related factors

**Fig. 9:**
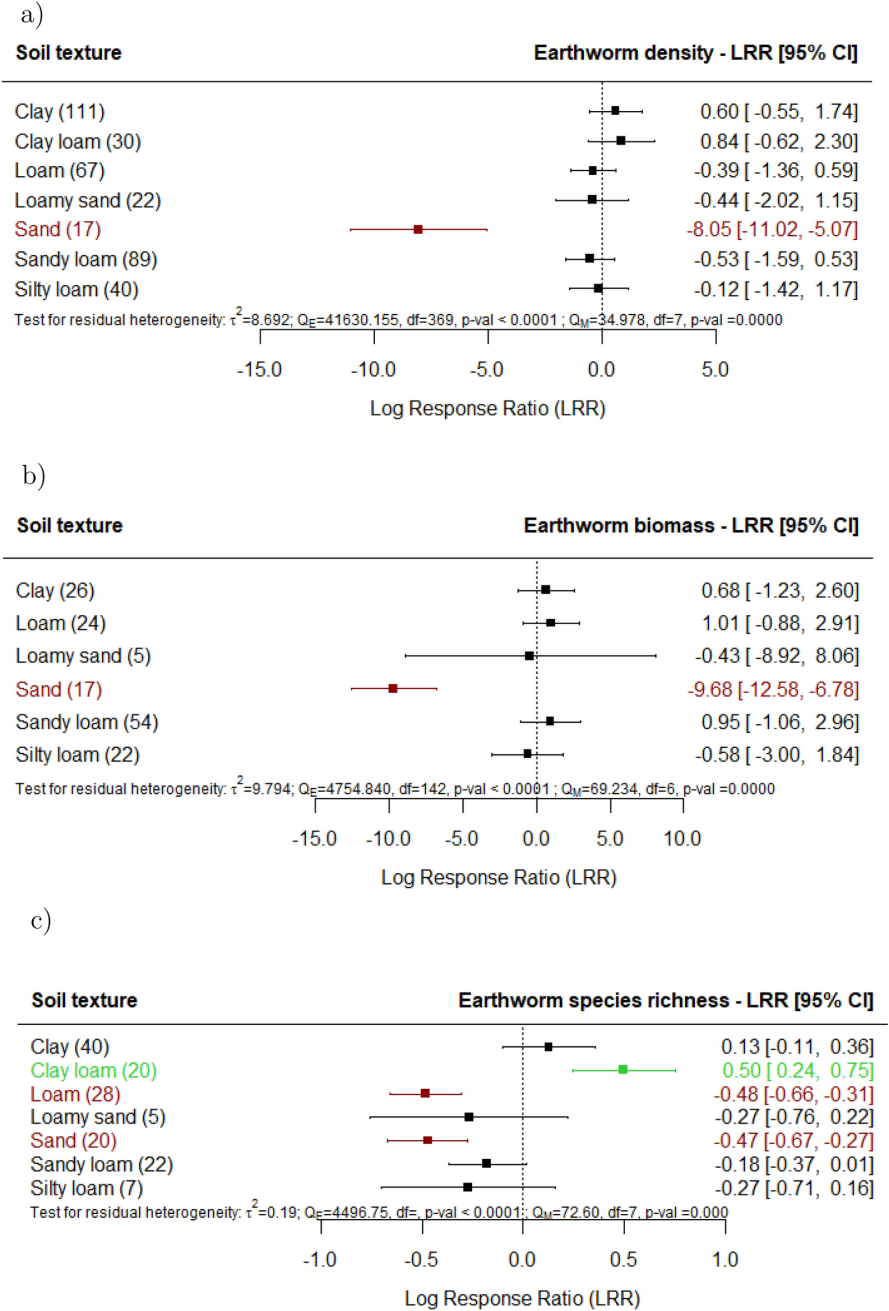
Effect of agricultural land use on earthworm (**a**) density, (**b**) biomass and (**c**) species richness, for the various soil textures documented in the studies in comparison with undisturbed sites (e.g., primary forest, undisturbed grasslands). The following forest plot illustrates the average effect (squares), with the 95% confidence intervals presented in brackets, and the sample sizes (number of pairwise comparisons) for each group enclosed in parentheses. Significantly negative effects are colored in red. Significantly positive effects are colored in green.

The assessment of the correlation between the effects of agricultural land use on earthworms and soil characteristics (Table 1) showed that in areas with higher soil compaction, the effects of agricultural land use on earthworm density were more pronounced, indicating that soil compaction potentially resulting from agricultural land use amplifies the impact on earthworm populations. Additionally, our findings demonstrated that in sites with higher soil organic matter content species richness is significantly higher in agricultural land use compared to undisturbed sites. Significantly lower earthworm density, biomass, and diversity reported as species richness and Shannon-Index (Supplement 3) were observed in agricultural land use in sandy soils (Fig. 9a-c). In loamy soils, we observed lower species richness in agricultural land use; in clay loams, higher species-richness was observed.

### Publication bias and heterogeneity

Publication bias was assessed independently for earthworm density, biomass, and diversity (see Supplement 5 for more details). Regarding density, the rank correlation test revealed significant funnel plot asymmetry ($p<0.0001$) despite the regression test producing a non-significant result ($p=0.6586$). On the other hand, for biomass, the rank correlation test revealed a substantial asymmetry in the funnel plot ($p<0.0001$), despite the regression test yielding a non-significant result ($p=0.6510$). Finally, the rank correlation test found substantial asymmetry ($p<0.0001$) in the assessment of diversity, but the regression test was unable to find funnel plot asymmetry ($p=0.6779$). In summary, although the rank correlation tests detected funnel plot asymmetry (suggesting possible bias), the regression tests did not confirm this. This discrepancy could mean that while there is some evidence of asymmetry in the data, it may not strongly indicate publication bias.

Heterogeneity characterizes the meta-analysis outcomes across various parameters, as evidenced by the Q-test results. For density, a thorough examination reveals significant heterogeneity (Q=1160172.8935, $p<0.0001$, $\tau ^2=6.9861$, $I^2=99.8519$%), indicating substantial variation in true outcomes among observations. Similarly, biomass exhibits pronounced heterogeneity (Q=606565, $p<0.0001$, $\tau ^2=9.8804$, $I^2=99.7835$%), suggesting diverse underlying processes driving biomass differences among studies. Diversity patterns also display significant heterogeneity (Q=83299, $p<0.0001$, $\tau ^2=0.3788$, $I^2=99.6910$%), reflecting varied dynamics within sampled communities. Additionally, substantial residual heterogeneity is observed across different agricultural land use, climatic, and soil-related factors, indicating contributions beyond random chance to effect size variability $(Q_E, p<0.001)$. Moreover, omnibus tests for variation among the different levels of agricultural land use, climatic and soil related factors assessed as predictors of variability, reveal significant differences in most cases $(Q_M,p<0.001)$. This highlights the complexity of the earthworm response to agricultural land use, and the importance of management, soil and climatic factors in determining earthworm outcomes.

## Discussion

The meta-analysis results underscore the significant influence of agricultural land use on earthworm populations. Notably, in arable cropland, particularly rotations in annually cropped agricultural fields, we observed substantially lower earthworm density, biomass, and species richness, in line with several primary studies conducted in different regions^26^. Conversely, in pasture, agroforestry, and crop management with fallow periods higher earthworm density was observed compared to undisturbed sites. This is probably driven by a greater quantity and variety of sources of organic matter^27^, or higher microbial efficiency in the transformation of organic matter into fractions that earthworms can assimilate^28^. The presence of exotic species may justify the higher biomass in agricultural land use since these earthworm species often colonize disturbed ecosystems, mostly in the tropics^29–31^. However, it is important to consider that the introduction of these exotic species may result in the replacement of native species, potentially altering the local ecosystem dynamics and biodiversity.

Much literature has described lower species richness under agricultural land use^22,27,32–38^, which reduces the potential population expansion when there is a limited supply of organic inputs. Compared to arable croplands, sites with more diversified agricultural land uses may still have some degree of habitat complexity and variability, which can support a wider variety of species^39^and thus increase regional earthworm diversity. Furthermore, some native species may persist in agricultural environments with minimal population decline if they are able to adapt to a certain level of disturbance from agricultural activities^40^. At locations with varying forms of agricultural land use, other than arable croplands, the specific species present can change due to turnover, which means that some species may disappear while new ones take their place. Despite these changes, the overall species richness may remain relatively stable. This implies that even though certain species are lost, they are replaced by others, maintaining a similar level of diversity. On the other hand, the lower species richness observed can be due to a population increase of opportunistic species^41^.

The agricultural land use effects in sites where combined mineral and organic fertilizers had been applied appeared to be detrimental on species richness. We should note that the arable cropland with intensive rotations and managed plantations^32,42–45^ were the corresponding to the studies in which the observations were made. In the individual studies that reported the effects of mixed organic and mineral fertilization, the loss of species richness was often attributed to lower soil temperatures and water content rather than specifically to the use of mixed fertilizers. As a result, the true effects of the mixed fertilizers on species richness could have been obscured by these other factors, potentially masking their direct impact.

Similarly, the absence of pesticide treatment correlated with higher earthworm density, whereas herbicide spraying resulted in reduced biomass and species richness. It is broadly documented that the use of glyphosate-containing herbicides, which was extensively used in the agricultural locations examined^46–50^, may potentially cause a decline in earthworm density, biomass and species richness. The positive effects of agricultural land use in sites where pesticides were not applied could also be masked by the use of field margins in areas under agricultural land use^51^.

Agricultural land use effects in arable cropland, even in sites undergoing intensive rotations, under no tillage showed consistent positive outcomes on earthworm density^59^. Due in part to the modifications that no-tillage makes to the physical, chemical, and physicochemical properties of the soil, agricultural land use under this farming method has a positive impact on earthworm density^61^. On the other hand, under reduced tillage the concurrent application of herbicides and the use of intensive rotations in these agricultural land uses may have contributed to the lower species richness^33,42,44^. This indicates that implementing a singular less intensive practices might not suffice to safeguard earthworm diversity. Instead, adopting multiple less intensive practices concurrently is essential for effectively preserving earthworm diversity.

Our study elucidated the role of climatic conditions in shaping earthworm responses to agricultural land use Locations with continental climates characterized by no dry season and cool summers exhibited higher earthworm density and biomass in agricultural land use. These positive effects may be explained by the fact that in these climatic zones, the studies documented the effects of agricultural land use as managed mixed grasslands^62–64^, and not to more intensive agricultural land use types. Species richness, a more sensitive parameter to agricultural land use effects across different climatic zones, shows that in regions with tropical rainforest climates and temperate climates with hot summers, agricultural land use had detrimental effects on earthworm diversity^26,33,34,36,42,64,65^. These areas might be hosts to distinctive diverse earthworm communities that are especially vulnerable to agricultural land use-related environmental changes^66^. It is possible that earthworm species in cold climates are more adapted to agricultural practices, as these environments have undergone human land-use changes for longer periods, allowing species to evolve or acclimate to disturbances^67^. In contrast, earthworm species in tropical zones may be less adapted to agricultural land use, potentially because these ecosystems have experienced less historical agricultural pressure^66^. These results demonstrate the complex relationship that exists between changes in land use, climate, and earthworm communities, implying that land management plans need to take regional climate variances into consideration.

Significant relationships between soil properties and earthworm reactions to agricultural land use were found using meta-regressions. Higher soil compaction was associated with greater detrimental agricultural land use effects on earthworm density^49,68–70^. Compacted soil restricts earthworm movement and burrowing activity^71^, and limits the availability of oxygen within the soil profile which can inhibit earthworm survival and reproduction^72^. Excessive compaction can lead to waterlogging in certain areas and drought stress in others^73^, creating unfavorable conditions for earthworms^74^. Therefore, higher soil compaction can further exacerbate the adverse effects of agricultural land use on earthworm density, making it more challenging for earthworm populations to persist in managed agricultural landscapes.

Higher organic matter content was linked to higher species richness observed consistently across different forms of agricultural land use^31,52,53,75–77^. In general, soils with higher organic matter content foster a more conducive environment for earthworms, by providing higher nutrient availability for diverse plant species to thrive, supporting a greater diversity of earthworm species^27^. By facilitating more root penetration, water infiltration, and air exchange, organic matter helps to improve soil structure, which in turn makes earthworm habitat more favorable^78^and may increase their ability to resist the negative effects of agricultural land use. Organic matter in the soil helps to maintain moisture in the soil and lowers the danger of drought stress^79^, supporting increased earthworm species diversity regardless of the type of agricultural land use. This is also consistent with our observations of the detrimental impacts of agricultural land use on the biomass, species richness, and density of earthworms in sandy soils^34,62^, which often have low organic matter contents. Agricultural land use in loamy soils was shown to have lower species richness as well, demonstrating that the application of herbicides in intensive rotations might have detrimental effects on earthworm diversity even in soils with more favorable textures for earthworms^22,23,31,33,44,80^.

The assessment of publication bias and heterogeneity in our meta-analysis revealed significant challenges in synthesizing data from a wide range of studies. Publication bias, which occurs when studies with statistically significant results are more likely to be published, was evident through significant funnel plot asymmetry for density, biomass, and diversity metrics. This suggests that studies reporting significant positive or negative effects of agricultural land use on earthworm populations may be overrepresented in the literature compared to studies with null or negative findings. Additionally, the regression tests conducted to assess publication bias yielded mixed results, further complicating the interpretation of our findings. This inconsistency underscores the need for rigorous statistical approaches to account for potential biases and uncertainties inherent in meta-analytic studies.

Furthermore, substantial heterogeneity was observed across various parameters, indicating further diverse underlying processes influencing earthworm responses to agricultural land use This heterogeneity may stem from variations in study methodologies, geographic locations, agricultural land-use practices, soil types, climatic conditions, and other factors. Such complexities underscore the importance of careful interpretation of meta-analytic results and the need for robust statistical techniques to account for heterogeneity. Despite our efforts to address these challenges, residual heterogeneity persisted in all factor analyses, indicating that additional unmeasured factors or the interactions may contribute to variability in earthworm responses. Interactions among the assessed factors, such as the combined effects of specific management practices, climatic conditions, and soil properties, could further explain this variability. However, due to data sparsity and limitations in the available literature, assessing these interactions with our current dataset was not feasible.

## Conclusion

Our meta-analysis provides compelling evidence of the profound impact of agricultural land use on earthworm populations across diverse agroecosystems. The detrimental effects observed in arable croplands with intensive rotations underscore the vulnerability of earthworm populations to disturbances such as tillage intensity, agrochemical use, soil organic matter content and soil compaction. However, certain agricultural land uses such as agroforestry and the use of fallow periods in combination with reduced agrochemical input demonstrate the potential to mitigate these negative impacts.

Furthermore, our research highlights the complex interactions that occur between earthworm responses to agricultural land use, soil characteristics, and climate. Regions with continental climates characterized by cool summers exhibit more favorable outcomes for earthworm populations, while excessive soil compaction and low organic matter content exacerbate the adverse effects of agricultural land use. To protect earthworm diversity and ecosystem function, strategies for land management must take into consideration regional climate variations and soil attributes. In essence, preserving earthworm populations is integral to sustainable land management practices, as these organisms play a significant role in soil health and ecosystem resilience. By implementing holistic approaches that prioritize habitat conservation, organic matter management, and reduced agrochemical inputs, we can mitigate the adverse effects of agricultural land use and promote earthworm diversity conservation in agricultural landscapes.

Although this meta-analysis provides thorough insights into how agricultural land use affects earthworms, the highly heterogeneous responses of earthworms to agricultural land use in global agroecosystems cannot be explained by the individual evaluation of climatic, soil-related, or management factors. If more data on agricultural land use effects were available for meta-analysis for example on the effects of crop livestock integration, field margins, and specialty crops, it might be possible to explain this variability through complex interactions among mentioned components. Future efforts should aim to address data limitations and enhance our understanding of the interactive effects of various factors on earthworm responses to agricultural land use. Long-term monitoring studies across diverse landscapes and climatic regions could provide a deeper understanding of temporal dynamics and ecosystem resilience. Furthermore, evaluating the effects of agricultural land use on particular species or analyzing the response of earthworm communities’ functional diversity and composition to agricultural land use may improve our comprehension of these organisms’ ecological roles and responses. Moreover, evidence-based land management strategies targeted at promoting sustainable agricultural systems while preserving soil biodiversity can benefit from multidisciplinary research combining soil science, ecology, and agronomy.

## Methods

### Search strategy

The following search engines were used: Scopus and Web of Science (Including the following databases: Web of Science Core Collection, KCI-Korean Journal Database, MEDLINE®, Preprint Citation Index, SciELO Citation Index) (Supplement 1). The starting point was an existing search carried out using a search summary table^81^, that contained detailed terms identified through the PICO approach (for more details on the search, screening, extraction and synthesis methods please refer to the OSF registration^82^). We included search terms such as agriculture, agroecosystems, land use, farmland, among several others detailed in Supplement 1. The inclusion criteria considered that studies reported: (i) density or biomass data, not only community composition in fields under different agricultural land uses; (ii) diversity metrics such as species richness, Shannon Index or Simpson’s diversity indices; (iii) means and sample sizes (number of pairwise comparisons) in control (undisturbed ecosystems i.e., undisturbed grassland, primary forest, secondary forest with more than 30 years of establishment) and treatment (different forms of agricultural land use ). Furthermore, studies with missing standard deviation or standard error were also included in the meta-analyses (Supplement 7). Studies should have data available in the form of text, tables or figures. When the data were presented as graphs, we manually digitized the figures to estimate means and standard deviation or standard error using WebPlotDigitizer Version 4.3. The different agricultural land use trials should have been conducted on the same site as the undisturbed reference ecosystem, to avoid additional variability due to soil properties and site characteristics. The soil of control and treatment have similar properties. If amendments were applied, these did not have high concentrations of heavy metals or cause potential toxicity to soil animals, i.e., studies with untreated sewage sludge or other types of industrial residues were not selected. The classification of agricultural land use types used for meta-analysis was conducted based on the information on the treatments reported in the individual articles, as follows:

- Agroforestry: Trees and shrubs incorporated within agricultural settings
- Crop-livestock integration: Refers to systems where both crop production and livestock grazing are integrated, often in a rotational system. This typically involves alternating phases of crop cultivation and grassland or pasture use on the same plot.
- Fallow: Faunal measurements in periods when agricultural land remained uncultivated and not actively managed.
- Field margins: Strips of vegetation left uncultivated, along the edges of agricultural fields established to enhance biodiversity, prevent soil erosion, and support pollinators and natural pest predators.
- Managed forest: Secondary forests and forests subject to levels of disturbance or human management.
- Managed grasslands: Intensively used grasslands, fertilized, subject to frequent cutting, and intensively mown areas, where grass is regularly harvested for fodder or other purposes.
- Managed plantations: Single or multiple species per plot cultivated for commercial purposes under controlled conditions, with the aim of producing timber, pulp, or other wood products.
- Orchard: Areas dedicated to the cultivation of fruitbearing trees or shrubs, managed using organic or conventional methods.
- Pasture: Grassland that is specifically managed for livestock grazing, modified through seeding, fertilization, or fencing.
- Plantation with specialty crop: Cocoa plantations, rubber monoculture, and mixtures like rubber and tea.
- Specialty crop: Specific types of agricultural product subject to specific management requirements, rather than for mass production or widespread consumption.
- Arable cropland: Wide range of cultivated areas and practices, including long-term arable fields and newly established plots. Both conventional and organic farming methods were included, with crops such as maize, soybean, rice, and beans grown either individually or in mixed cropping systems. Various tillage techniques, including conventional and reduced tillage were included. No tillage systems were also considered. Additionally, horticulture, sugarcane plantations, and meadows in rotation contribute to the diversity of agricultural land uses.

In addition, the different cropping systems found within the arable cropland classification were analyzed separately in the meta-analysis and categorized as follows:

- Rotation: Systematic rotation of crops in a specific sequence over time on the same piece of land.
- Monoculture: Cultivation of a single crop species over a large area of land.
- Intercropping: Growing two or more crop species simultaneously in the same field.
- Polyculture: Extends the concept of intercropping by involving the cultivation of multiple crop species together in a more complex and diverse system.

### Meta-analysis methods

The response ratio was used as the outcome metric in the analysis and a hierarchical correlated effects model was fitted. With sample sizes of $n_{iT}$ and $n_{iC}$, each of the $k$ studies that contributed to the meta-analysis had two arms: treatment ($T$) and control ($C$). In study $i$, the total sample size was $n_i=n_{iT}+n_{iC}$. Each arm’s subject-level data was assumed to have means of $\mu _{iT}$ and $\mu _{iC}$, variances $\sigma _{iT^2}$ and $\sigma _{iC^2}$, and distributions that were either log-normally distributed or normally distributed. For $i=1,...,k$ and $j=C or T$, the sample variances were $s_{ij^2}$, and the sample means were $\overline{X_{ij}}$. The response ratio was meta-analyzed on a log scale, with the population and sample means assumed to be positive, and the effect measure being $\lambda _i= log(\mu _{iT}/\mu _{iC})$, approximated by ${\hat{\lambda }}_i= log(\overline{X_{iT}}/\overline{X_{iC)}}$.

The restricted maximum-likelihood estimator was utilized to estimate the level of heterogeneity, or $\tau ^2$^83^. The Q-test for heterogeneity^84^ and the $I^2$statistic^85^ are presented in addition to the estimate of $\tau ^2$. A prediction interval for the real outcomes is also given, in the event that any degree of heterogeneity was found (i.e., $\tau ^2>0$, independent of the Q-test findings)^86^. To investigate whether studies might represent outliers or are significant within the framework of the model (details given below), studentized residuals and Cook’s distances were employed^87^. Research articles with a studentized residual greater than the $100*(1-0.05/(2*k))^{th}$ percentile of a standard normal distribution were deemed to be probable outliers; for k studies that were part of the meta-analysis, this means using a Bonferroni correction with a two-sided $\alpha =0.05$. Studies were deemed to be important if their Cook’s distance was more than the median plus six times the interquartile range. To assess for funnel plot asymmetry, two methods were used: the rank correlation test^88^and the regression test^89^, which uses the standard error of the observed outcomes as predictor. R (version 4.3.2)^90^ and the *metafor*package (version 4.4.0)^91^ were used for the analysis.

Our meta-analysis additionally included studies that supplied mean values and number of replicates for experimental and control groups, but had missing precision measurements (because of incomplete reporting or low-quality statistics). A meta-analysis including studies with missing precision data is nevertheless useful, and disregarding such research could lead to bias. Using the R package *mice*^92^, multiple imputation using chain equations was used to impute the missing variances from individual studies (sensitivity analysis in Supplement 6). Regression trees and classification were applied as a univariate imputation technique. Using the *pool* function of the *mice* package, which aggregates the estimates from repeated complete data analysis using Rubin’s combination rules, the estimated effect sizes and variances are pooled estimates of 20 imputed datasets.

Due to reporting of numerous follow-up times, distinct treatment conditions with a shared control, measurements at different depths, or different within-site locations, the majority of studies report multiple outcomes that reflect dependent data. As a result, we used the correlated and hierarchical effects model for our meta-analyses. This model combines correlated effect sizes within studies with dependent patterns that result from a multilevel data structure (i.e., observations within studies). We assumed the sample correlation between the effect sizes within a study to be constant (sensitivity analysis in the Supplement 6). We believed that a hierarchical structure was necessary since there was cause for within-study variation in the genuine impact sizes. The *clubSandwich* and *metafor* R packages were used in conjunction to accomplish this modeling strategy. Within each study, the correlation structure of the effect-size estimations are specified using the *impute_covariance_matrix()* function of the *clubSandwich*package^93^. Next, we created constrained maximum likelihood estimates of the random effects and the meta-regression coefficients using the *rma.mv()* function of the *metafor *package^87^.

The mean density, biomass, or diversity of earthworms are the response variables selected for investigation. To specify the hierarchical structure of the data (sensitivity analysis in Supplement 6), mixed-effects meta-regressions were performed with the following moderators: climate (mean annual precipitation, climatic zone), soil properties (texture, pH, organic matter content, organic carbon content, bulk density), and management practices (tillage method, concurrent application of herbicide and fertilizer, period under the studied land use). As our reference ecosystems are natural, undisturbed sites, the lack of significant results implies that earthworm populations appear to be mostly unaffected by agricultural land use, according to these control conditions. However, these non-significant effects should be interpreted with caution due to high levels of residual heterogeneity for most factors (see below). Observation and study identifier were included as random-effects in the meta-regressions.In addition to the between-study variance $\tau ^2$, we estimated the residual heterogeneity $(Q_E)$ observed across different land-use, climatic, and soil-related factors that moderate earthworm responses to agricultural land use. A significant $(Q_E, p<0.05)$ would indicate contributions beyond random chance to effect-size variability. We also conducted between-group heterogeneity tests $(Q_M)$ to compare the responses of earthworm density, biomass and diversity to land use among the different subgroup responses for a given moderator. A significant $(Q_M,p<0.05)$ indicated that the response ratios differed among categorical factors. Moreover, omnibus tests for variation among the different levels of the agricultural land use, climatic and soil related factors assessed as predictors of variability, reveal significant differences in most cases $(Q_M,p<0.001)$.

**Table 1:** Effect of agricultural land use on earthworm density, biomass and species richness in relation to the soil pH, organic matter content and bulk density documented in the studies. The regression coefficients, standard error and $p-values$ are shown for each response variable. More details on the meta-regressions can be found in the supplement 4 for pH, organic matter and bulk density.

Soil property	Response	Coefficient	Std. error	$p-value$
pH	Density	−0.06	0.20	0.78
	Biomass	−1.29	3.11	0.68
	Species richness	−0.01	0.07	0.89
Organic matter	Density	0.02	0.03	0.50
	Biomass	−0.77	0.76	0.32
	Species richness	0.04	0.02	0.03
Bulk density	Density	−0.35	0.15	0.02
	Biomass	2.06	1.43	0.15
	Species richness	−0.13	0.08	0.10

## Supplementary Information

Supplementary Information 1.

## Data Availability

The data that support the findings of this study is openly available at: https://doi.org/10.5281/zenodo.10809839. This meta-analysis was registered at the Open Science Framework (OSF) to ensure transparency and reproducibility following the structure of the PRISMA (Preferred Reporting Items for Systematic Reviews and Meta-Analyses) checklist (Supplement 7). The pre-registration can be accessed at: https://doi.org/10.17605/OSF.IO/XYMFJ.
