# Supplementary material for: The impact of multiple agricultural land uses in sustaining earthworm communities in agroecosystems - A global meta-analysis
Source: Sci Rep. 2024 Dec 4;14:30160. doi: 10.1038/s41598-024-81676-5 (PMC11615216; doi:10.1038/s41598-024-81676-5)
Supplement: Supplementary file 1 — Supplementary Information 1. [file 41598_2024_81676_MOESM1_ESM.pdf]

Supplementary material - The impact of multiple agricultural land uses in sustaining earthworm communities in agroecosystems: A global meta-analysis

Contents

Supplement 1: Search string used to retrieve studies. **3**

Supplement 2: List of locations where each study was conducted **4**

Supplement 3. Intensification effects on earthworm diversity reported as Shannon Index (H') **11**
- Intensification form . . . 11
- Cropping system . . . 11
- Period of intensified use . . . 12
- Types of fertilizer . . . 12
- Types of pesticide . . . 13
- Mean annual precipitation . . . 13
- Climatic zone . . . 14
- Soil pH . . . 14
- Soil bulk density . . . 15
- Soil texture . . . 16
- Earthworm extraction methods . . . 16

Supplement 4. Additional meta-analyses on management, soil and climatic related factors that can affect earthworm response to intensification **17**
- Period of use . . . 17
- Mean annual precipitation . . . 19
- Soil pH . . . 21
- Soil organic matter . . . 23
- Soil bulk density . . . 25
- Earthworm extraction method . . . 27
- Estimated effects of management and climatic zone separately for each land use . . . 29

Supplement 5: Meta-analysis diagnostics **34**
- Earthworm density . . . 34

Earthworm biomass . . . 34
Earthworm diversity . . . 34

Supplement 6. Sensitivity analyses **35**
Within-study correlations . . . 35
Random-effects hierarchical structure . . . 35
Multivariate imputation . . . 37

Supplement 7. PRISMA flow diagram. *n* refers to the number of research articles and *obs* to the number of pairwise observations **45**

Supplement 8. Intensification effects on earthworm density per study **46**

Supplement 9. Intensification effects on earthworm biomass per study **49**

Supplement 10. Intensification effects on earthworm species richness per study **51**

References **51**

Supplement 1: Search string used to retrieve studies.

TI = (((agricultur* OR agroecosys* OR farm* OR farmland* OR field OR ((land* OR agr) NEAR use) OR crop* OR graz* OR soil) NEAR (manag OR system* OR practic* OR input* OR intens* OR gradient)) AND (biodiversity OR biological OR biota OR earthworm OR fauna OR invertebrate* OR lumbric* OR worm* OR oligochaet* OR annelid)) OR AB = (((agricultur OR agroecosys* OR farm* OR farmland* OR field OR ((land* OR agr) NEAR use) OR crop* OR graz* OR soil) NEAR (manag OR system* OR practic* OR input* OR intens* OR gradient)) AND (biodiversity OR biological OR biota OR earthworm OR fauna OR invertebrate* OR lumbric* OR worm* OR oligochaet* OR annelid) OR KP = (((agricultur OR agroecosys* OR farm* OR farmland* OR field OR land* OR crop* OR graz* OR soil) NEAR (manag OR use* OR system* OR practic* OR input* OR intens* OR gradient)) AND (biodiversity OR biological OR biota OR earthworm OR fauna OR invertebrate* OR lumbric* OR worm* OR oligochaet* OR annelid*) and Articles (Document Types) and Agriculture OR Environmental Sciences OR Ecology OR Plant Sciences OR Zoology OR Biodiversity Conservation (Research Areas)

Supplement 2: List of locations where each study was conducted

Reference	Continent	Country	Location
1	South America	Brazil	Pinheiro Machado
2	Europe	France	Lusignan
3	South America	Venezuela	Puerto Ayacucho
4	East Africa	Kenya	Embu, Kabete, Impala, Nyabeda
4	East Africa	Malawi	Chitala
4	West Africa	Nigeria	Ibadan
4	West Africa	Ghana	Tamale
4	West Africa	Niger	Sadore
4	West Africa	Burkina Faso	Farakoba, Saria
5	South America	Brazil	Embrapa Amazonia Ocidental
6	South America	Brazil	The West, The Plateau
7	South America	Argentina	Cordoba province
8	South America	Argentina	Monte Buey, Cordoba province
9	Asia	India	Camoli
10	Europe	Scottland	
11	South America	Brazil	Parana
12	North America	Canada	Southern Ontario and Southern Quebec
12	Europe	Czech Republic	South Bohemian region
13	Australia	Australia	New South Wales
14	Europe	France	Brittany
15	Central America	Costa Rica	Pueblo Nuevo de villa Franca de Guacimo, in Limon Province
16	Asia	Indonesia	Bodogol, Situ Gunung

Reference	Continent	Country	Location
[17]	South America	Peru	Quilcas, near Huancayo in the Junin
[18]	South America	Colombia	Carimagua “Altillanura Plana”
[19]	South America	Colombia	Oriental Llanos
[20]	Europe	France	Saint Martin de Boscherville, Upper Normandy
[21]	South America	Brazil	Lapa county (near Curitiba), Parana
[22]	South America	Brazil	Curitiba metropolitan area
[23]	South America	Argentina	Gral. Deheza basin, Cordoba
[24]	South America	Argentina	General Deheza basin
[25]	South America	Argentina	General Dehezabasin, Cordoba Cordoba province
[26]	South America	Argentina	South of Cordoba province, Buenos Aires Province and Chivilcoy
[27]	Europe	Germany	Wadrill valley and the Blies valley
[28]	Asia	China	Daqiao
[29]	South America	Colombia	Alcala, Cartago, Circasia, Filandia y Quimbaya.
[30]	South America	Colombia	Alcala Valle del Cauca
[31]	Europe	Germany	Trier

Reference	Continent	Country	Location
[32]	Europe	Germany	Kenner Flur, Halle (Saxony-Anhalt), Trier (Rhineland-Palatinate)
[33]	South America	Peru	Peruvian Amazonia
[34]	Africa	Ivory Coast	Lamto
[34]	Asia	India	Southern Karnataka; Western Chats, Karnataka
[35]	South America	Brazil	Jatai (Goias), Valparaiso and Ipaussu (Sao Paulo)
[36]	Oceania	New Zealand	Winchmore Irrigation Research Station in the mid-Canterbury region
[37]	Asia	China	Yellow Sea, Jiangsu
[38]	Central America	Mexico	Teapa
[39]	Central America	Puerto Rico	Luquillo Experimental Forest
[40]	Europe	Netherlands, Sweden, United Kingdom, Czech Republic	
[41]	South America	Uruguay	Los Molles, Tacuarembo; Joanico, Canelones; Ciudad de Treinta y Tres; Treinta y Tres Orientales, Florida; Melilla, Montevideo;
[42]	Europe	Italy	South Tyrol

Reference	Continent	Country	Location
[43]	Africa	Ivory coast	Centre-West Region
[44]	Oceania	New Zealand	Pukekohe; Winchmore Irrigation Research Station, Mid-Canterbury
[45]	Africa	South Africa	Baynesfield Estate, the KwaZulu-Natal midlands; Cedara Agricultural College/Research Station, the KwaZulu-Natal midlands
[46]	North America	United States	Georgia Piedmont
[47]	Central America	Mexico	Paso del Puente de Santa Ana, Puebla
[48]	South America	Brazil	Experimental areas of the Brazilian research institute Embrapa-Amazonia Ocidental
[49]	North America	United States	Sanborn field, University of Missouri
[50]	Asia	India	Narayankoti
[51]	North America	Mexico	Puebla; Oaxaca
[52]	Africa	Cameroon	Adamawa, North, Far-North

Reference	Continent	Country	Location
53	Europe	Slovakia	Nacina Ves, Voderady, Dvorniky, Moravsky Jan, Kecovo, Tajov, Liptovska Teplicka
54	North America	Canada	Southern Quebec
55	South America	Brazil	Chapeco
56	Europe	France	Western Brittany
57	South America	Peru	Yurimaguas Experimental Station in the Peruvian Amazonia
58	Asia	Pakistan	the district Sargodha
59	Europe	Spain	Tierras de Granadilla district; Central Western Spain (Tierras de Granadilla district)
60	Asia	India	Nilgiri biosphere reserve of Western Ghats
61	Africa	Cameroon	Fako Division in the Southwest region of Cameroon
62	South America	Brazil	Region of Jaguapita, Parana State
63	South America	Brazil	Ilha do Careiro; Embrapa Agropecuaria Oeste, em Dourados, MS
64	North America	United States	Central and Eastern Maine
65	Europe	Italy	Vallevecchia

Reference	Continent	Country	Location
[66]	Europe	France	Brittany, Quimper site, The Rennes site
[67]	Europe	Portugal	Rolandia
[68]	Europe	France	RMQS network
[69]	Europe	Netherlands	De Bovenbuurt
[70]	Europe	Netherlands	De Bovenbuurt
[71]	Europe	United Kingdom	North Yorkshire
[72]	Asia	India	Kiccha, Haldwani, Ranibag, Dogaon, Jeolikot
[73]	Africa	Madagascar	Antsirabe
[74]	Asia	India	Sagar Forest Range, Shimoga Division, Karnataka State
[75]	Europe	Italy	South Tyrol
[76]	South America	Colombia	Parque Regional Natural Ucumari y el Santuario de Flora y Fauna Otun-Quimbaya
[77]	South America	Brazil	Embrapa Agropecuaria Oeste, municipio de Dourados-MS; Vereda dos Anacletos, localizada no municipio de Esperantina
[78]	Asia	India	Village Neshta, Village Lopoke, Ajnala (Punjab); Assam, northeast India
[79]	North America	United States	SW Michigan
[80]	Europe	Austria	Kaserstattalm
[81]	South America	Colombia	Western Colombian Amazon

Reference	Continent	Country	Location
[82]	Europe	Portugal	Sao Miguel Island (Azores)
[83]	Africa	Ivory Coast	Oume Region
[84]	Africa	Ivory Coast	Foret Classee de la Tene
[85]	Europe	Sweden	Scania
[85]	Europe	United Kingdom	Region Chilterns
[85]	Europe	Czech Republic	Ceske Budejovice
[85]	Europe	Greece	Kria Brisi
[86]	Europe	Latvia	Dobele, Priekuii
[87]	Europe	Slovenia	University Maribor
[88]	Asia	Malaysia	Jengka Triangle
[89]	Oceania	New Zeland	Manawatu
[90]	South America	Uruguay	Ciudad de Treinta y Tres
[91]	North America	Puerto Rico	Sabana

Supplement 3. Intensification effects on earthworm diversity reported as Shannon Index (H')

Intensification form

Effect of intensified land use on earthworm diversity reported as Shannon Index (H'), for the various forms of intensification documented in the studies. Forest plot illustrates the average effect, with the 95% confidence intervals presented in brackets, and the sample sizes for each group enclosed in parentheses.

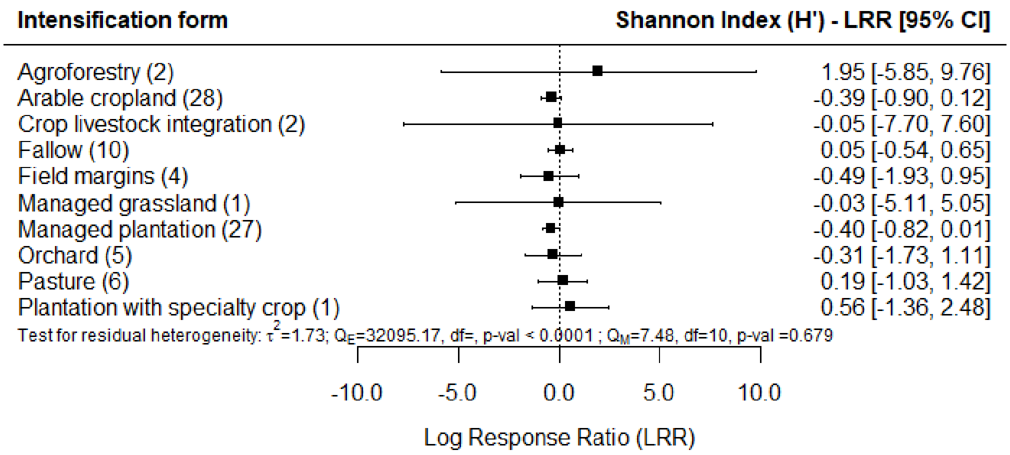

Cropping system

Effect of intensified land use on earthworm diversity reported as Shannon Index (H'), for the various cropping systems documented in the studies. Forest plot illustrates the average effect, with the 95% confidence intervals presented in brackets, and the sample sizes for each group enclosed in parentheses.

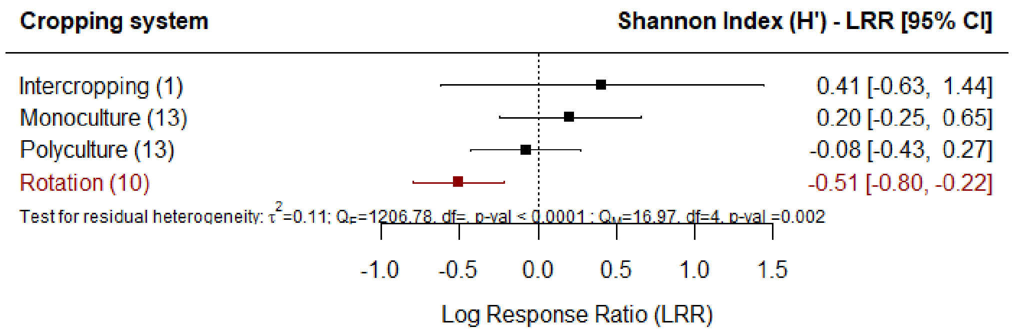

Period of intensified use

Effect of intensified land use on earthworm diversity reported as Shannon Index (H') in relation to the period of intensified use documented in the studies. The size of a regression bubble indicates the weight of each observation in the model and displays the mean effects. Gray, dotted lines indicate the bounds of the confidence interval over the regression line.

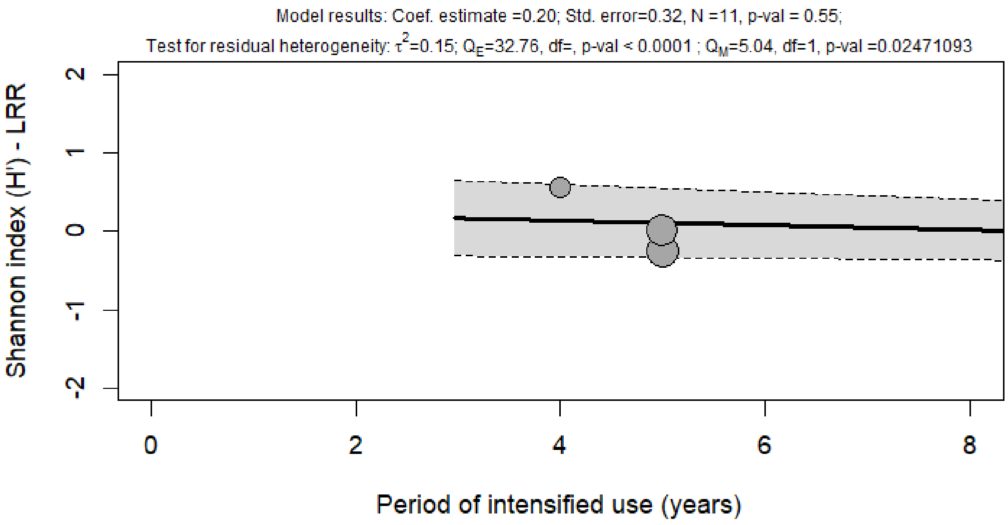

Types of fertilizer

Effect of intensified land use on earthworm diversity reported as Shannon Index (H'), for the various types of fertilizer documented in the studies. Forest plot illustrates the average effect, with the 95% confidence intervals presented in brackets, and the sample sizes for each group enclosed in parentheses.

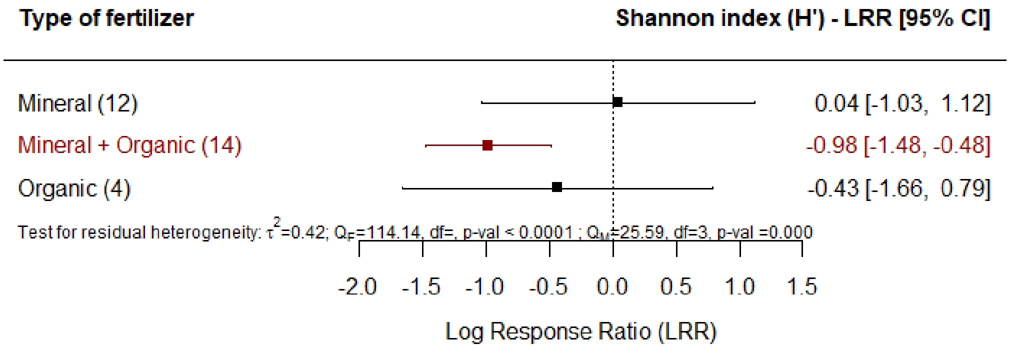

Types of pesticide

Effect of intensified land use on earthworm diversity reported as Shannon Index (H'), for the various types of pesticide documented in the studies. Forest plot illustrates the average effect, with the 95% confidence intervals presented in brackets, and the sample sizes for each group enclosed in parentheses.

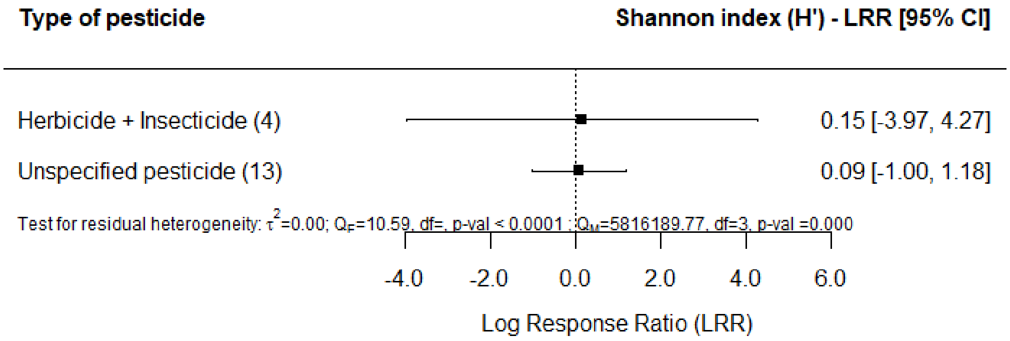

Mean annual precipitation

Effect of intensified land use on earthworm diversity reported as Shannon Index (H') in relation to the mean annual precipitation documented in the studies. The size of a regression bubble indicates the weight of each observation in the model and displays the mean effects. Gray, dotted lines indicate the bounds of the confidence interval over the regression line.

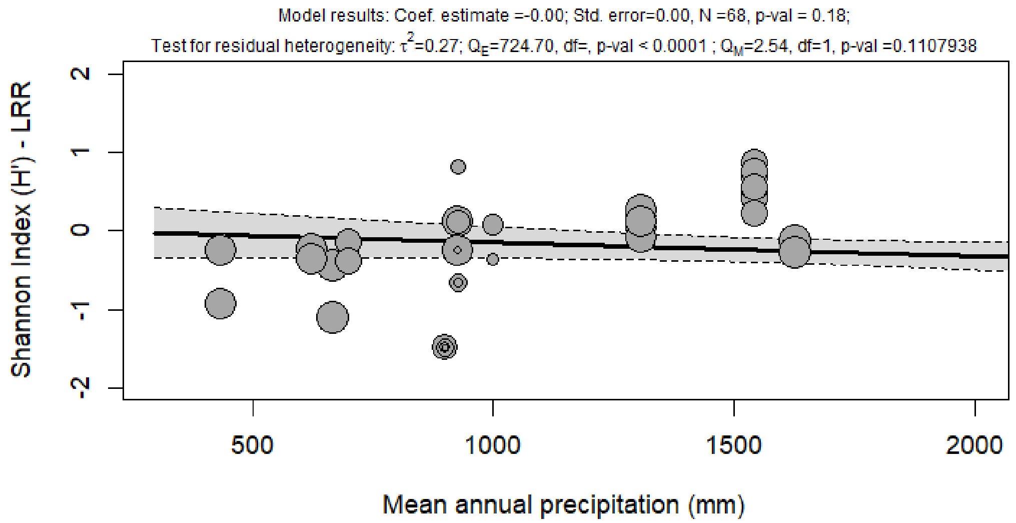

Climatic zone

Effect of intensified land use on earthworm diversity reported as Shannon Index (H'), for the various climatic zones documented in the studies. Forest plot illustrates the average effect, with the 95% confidence intervals presented in brackets, and the sample sizes for each group enclosed in parentheses.

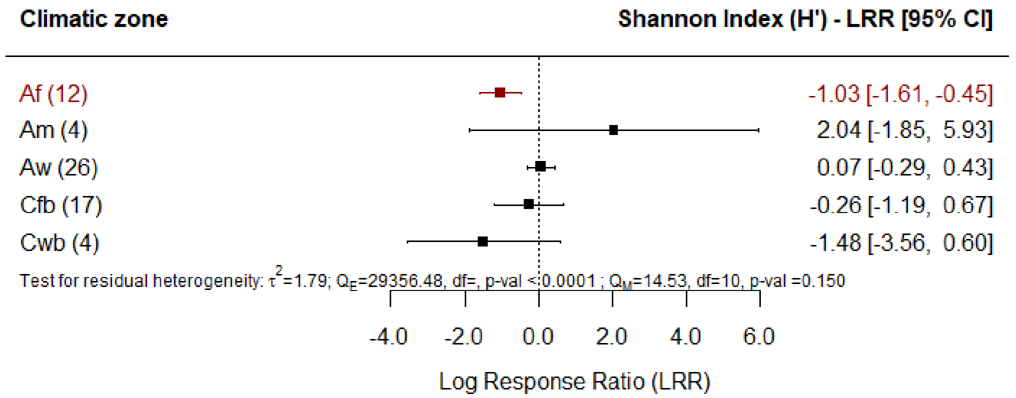

Soil pH

Effect of intensified land use on earthworm diversity reported as Shannon Index (H') in relation to the soil pH documented in the studies. The size of a regression bubble indicates the weight of each observation in the model and displays the mean effects. Gray, dotted lines

indicate the bounds of the confidence interval over the regression line.

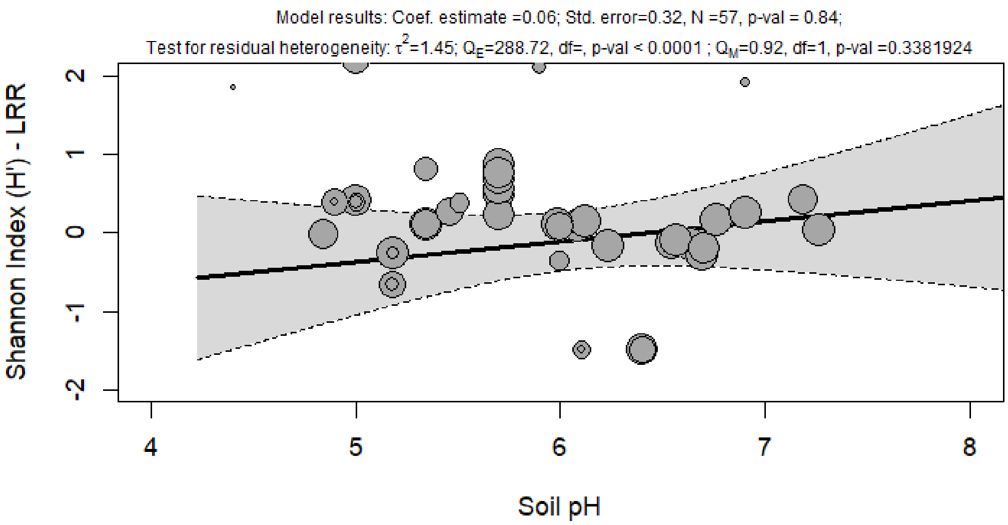

Soil bulk density

Effect of intensified land use on earthworm diversity reported as Shannon Index (H') in relation to the soil bulk density documented in the studies. The size of a regression bubble indicates the weight of each observation in the model and displays the mean effects. Gray, dotted lines indicate the bounds of the confidence interval over the regression line.

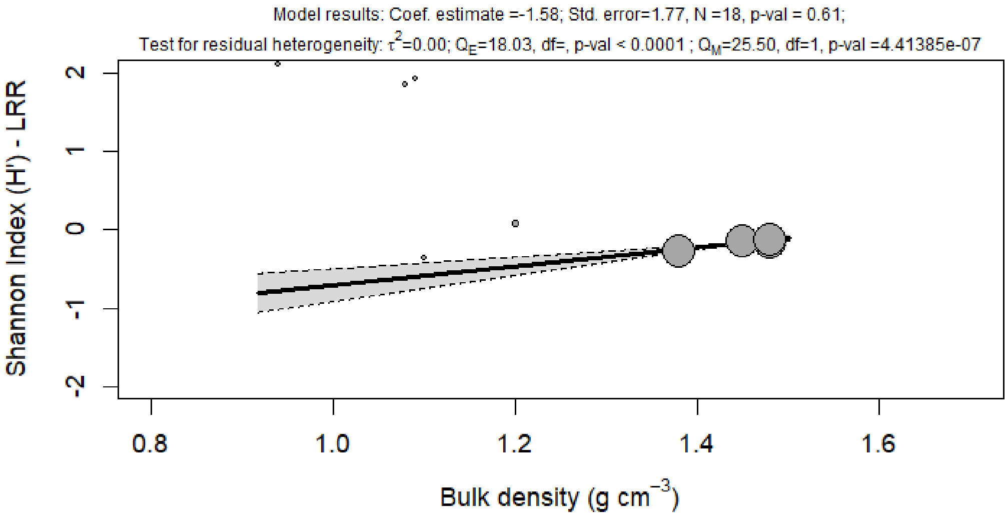

Soil texture

Effect of intensified land use on earthworm diversity reported as Shannon Index (H'), for the various soil textures documented in the studies. Forest plot illustrates the average effect, with the 95% confidence intervals presented in brackets, and the sample sizes for each group enclosed in parentheses.

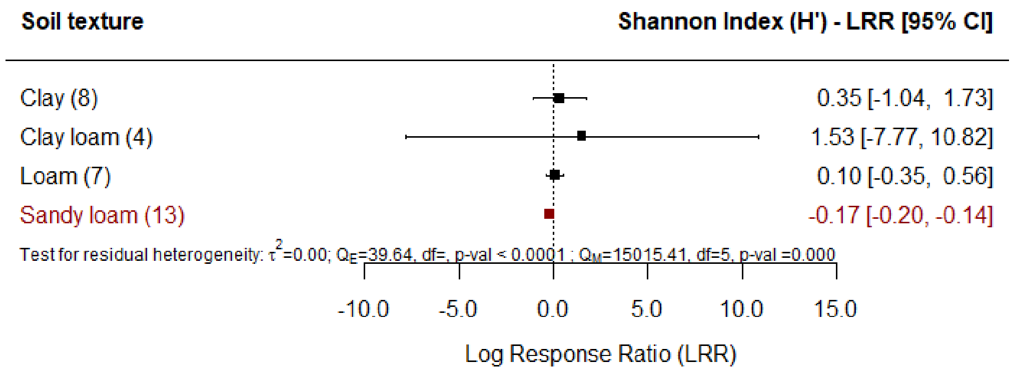

Earthworm extraction methods

Effect of intensified land use on earthworm diversity reported as Shannon Index (H'), for the various earthworm extraction methods documented in the studies. Forest plot illustrates the average effect, with the 95% confidence intervals presented in brackets, and the sample sizes for each group enclosed in parentheses.

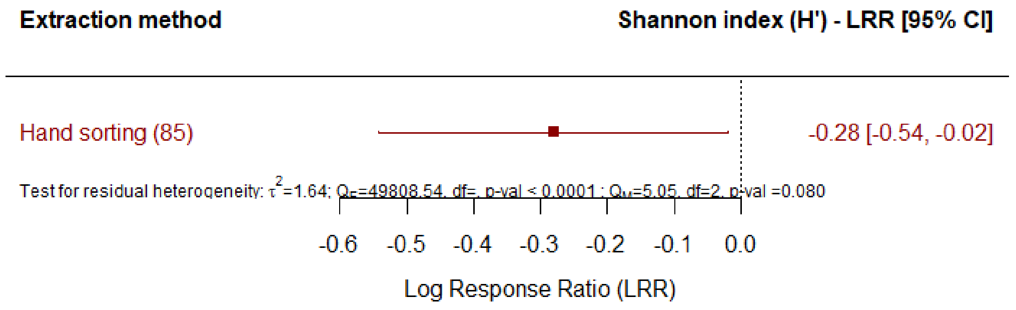

Supplement 4. Additional meta-analyses on management, soil and climatic related factors that can affect earthworm response to intensification

Period of use

Effect of intensified land use on earthworm a) density, b) biomass and c) species richness in relation to the soil period of intensified use documented in the studies. The size of a regression bubble indicates the weight of each observation in the model and displays the mean effects. Gray, dotted lines indicate the bounds of the confidence interval over the regression line.

a)

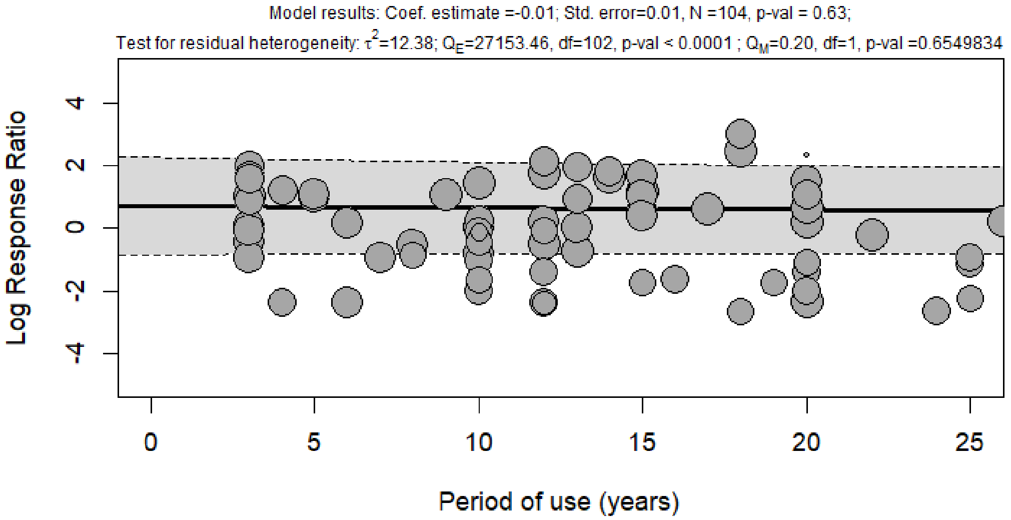

b)

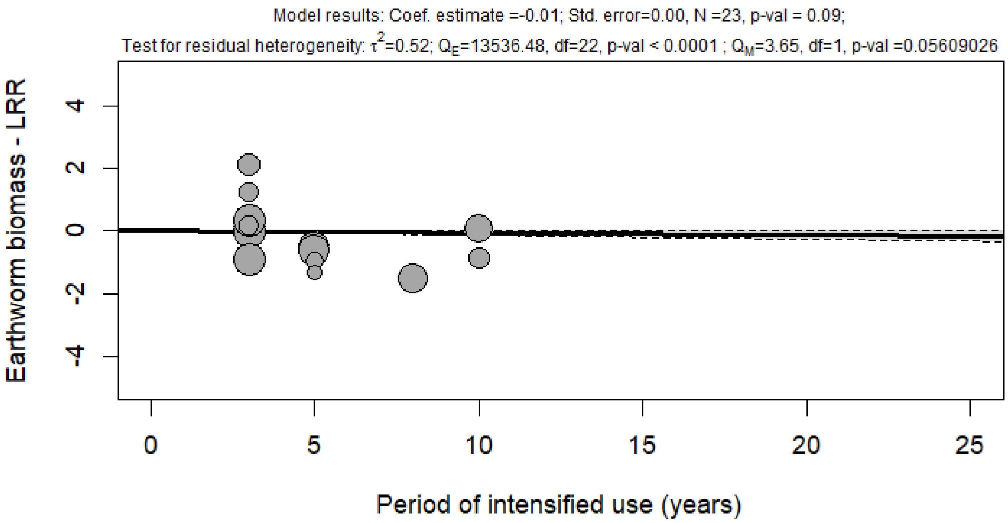
Model results: Coef. estimate =-0.01; Std. error=0.00, N =23, p-val = 0.09;
Test for residual heterogeneity: τ2=0.52; QE=13536.48, df=22, p-val < 0.0001 ; QM=3.65, df=1, p-val =0.05609026
Earthworm biomass - LRR
4
2
0
-2
-4
0
5
10
15
20
25
Period of intensified use (years)

c)

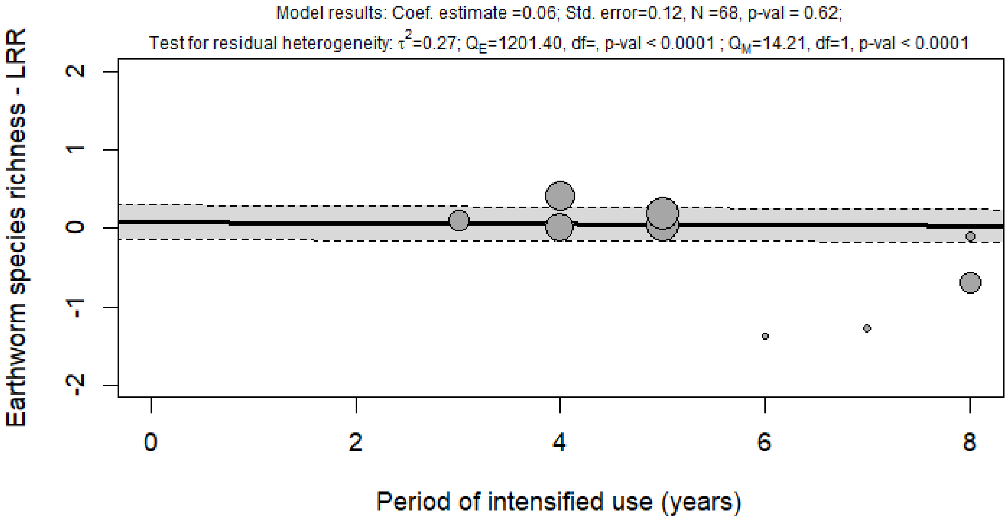
Model results: Coef. estimate =0.06; Std. error=0.12, N =68, p-val = 0.62;
Test for residual heterogeneity: τ2=0.27; QE=1201.40, df=, p-val < 0.0001 ; QM=14.21, df=1, p-val < 0.0001
Earthworm species richness - LRR
2
1
0
-1
-2
0
2
4
6
8
Period of intensified use (years)

Mean annual precipitation

Effect of intensified land use on earthworm a) density, b) biomass and c) species richness in relation to the mean annual precipitation documented in the studies. The size of a regression bubble indicates the weight of each observation in the model and displays the mean effects. Gray, dotted lines indicate the bounds of the confidence interval over the regression line.

a)

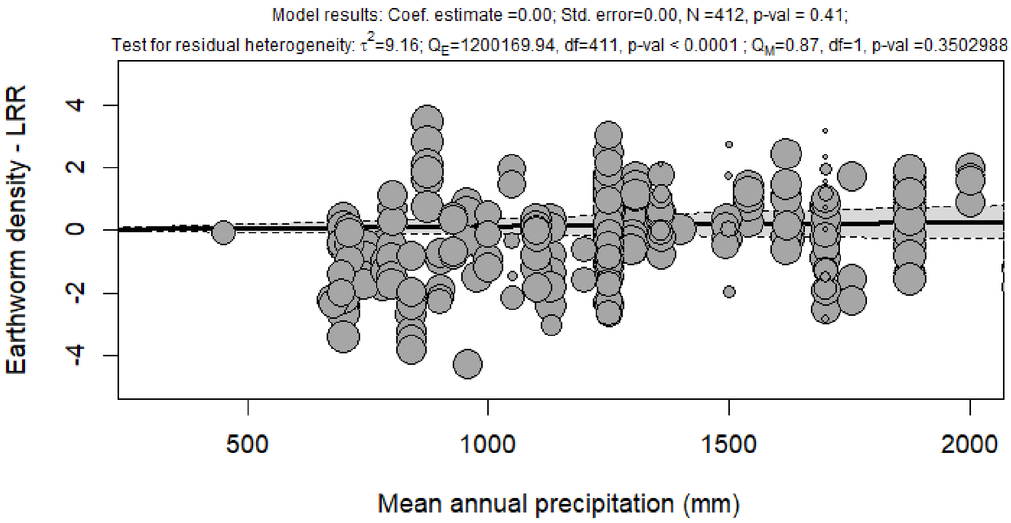

b)

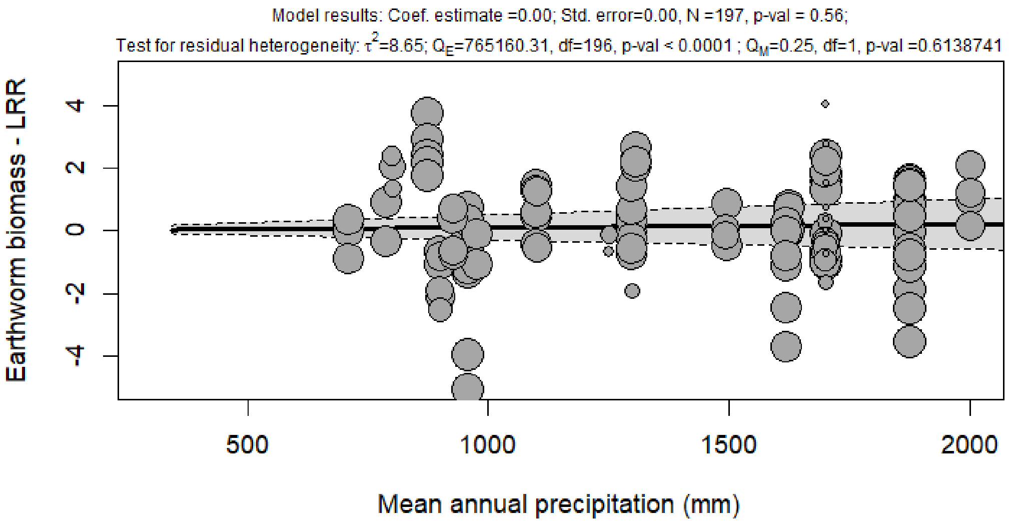

c)

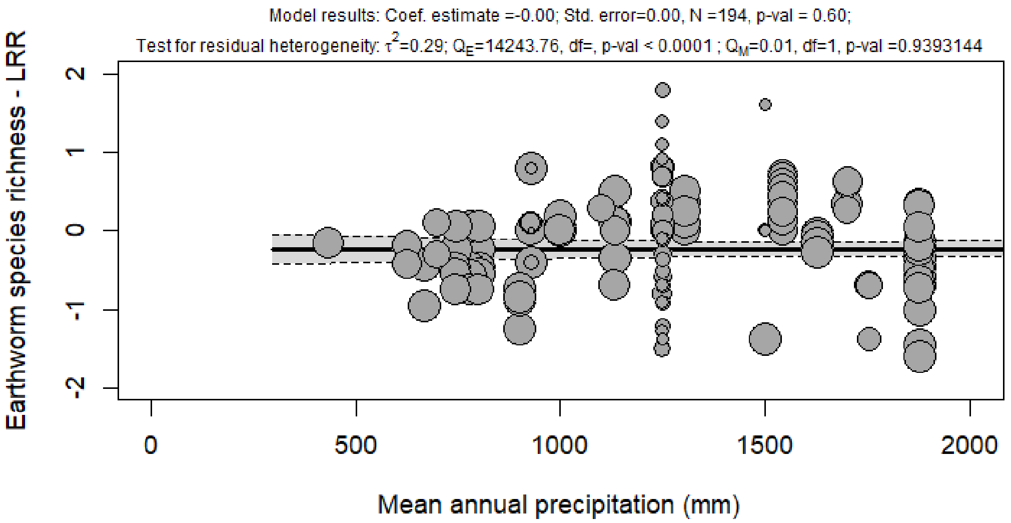
Model results: Coef. estimate =-0.00; Std. error=0.00, N =194, p-val = 0.60;
Test for residual heterogeneity: τ^2=0.29; Q_E=14243.76, df=, p-val < 0.0001 ; Q_M=0.01, df=1, p-val =0.9393144
Earthworm species richness - LRR
2
1
0
-1
-2
0
500
1000
1500
2000
Mean annual precipitation (mm)

Soil pH

Effect of intensified land use on earthworm a) density, b) biomass and c) species richness in relation to the soil pH documented in the studies. The size of a regression bubble indicates the weight of each observation in the model and displays the mean effects. Gray, dotted lines indicate the bounds of the confidence interval over the regression line.

a)

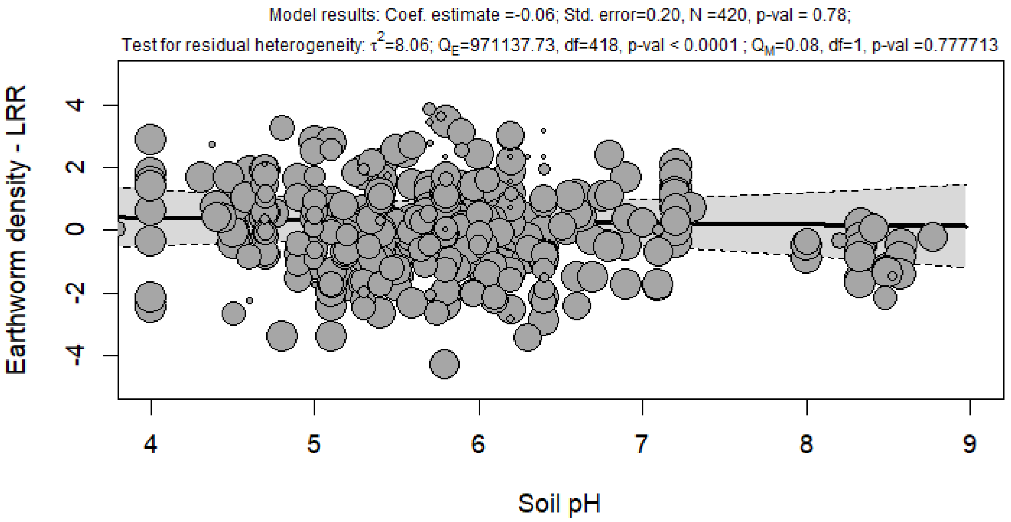

b)

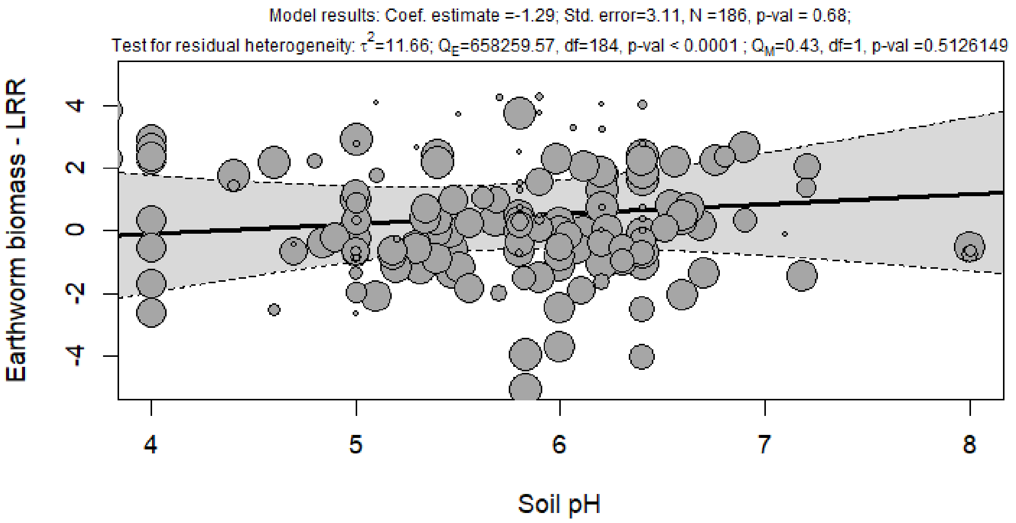

c)

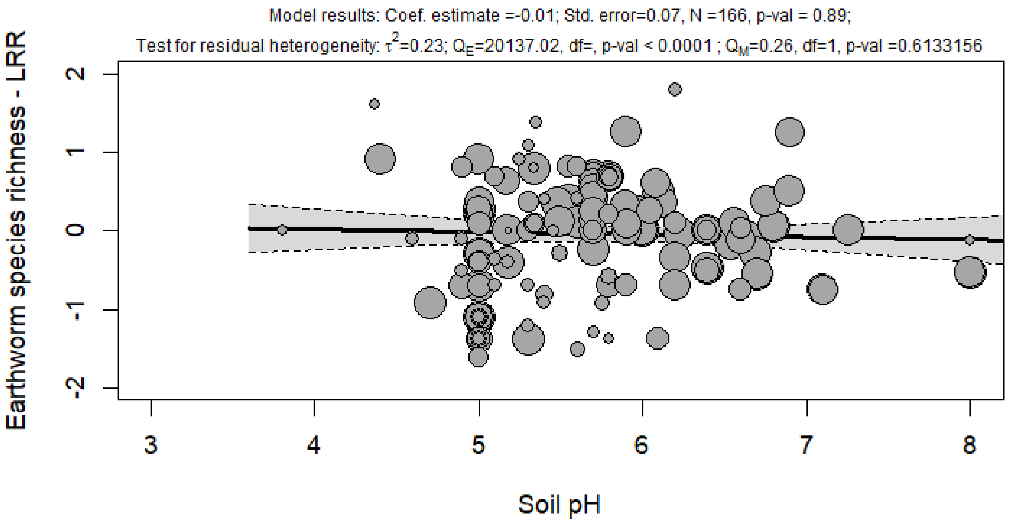
Model results: Coef. estimate =-0.01; Std. error=0.07, N =166, p-val = 0.89;
Test for residual heterogeneity: τ²=0.23; Q_E=20137.02, df=, p-val < 0.0001 ; Q_M=0.26, df=1, p-val =0.6133156
Earthworm species richness - LRR
2
1
0
-1
-2
3
4
5
6
7
8
Soil pH

Soil organic matter

Effect of intensified land use on earthworm a) density, b) biomass and c) species richness in relation to the soil organic matter documented in the studies. The size of a regression bubble indicates the weight of each observation in the model and displays the mean effects. Gray, dotted lines indicate the bounds of the confidence interval over the regression line.

a)

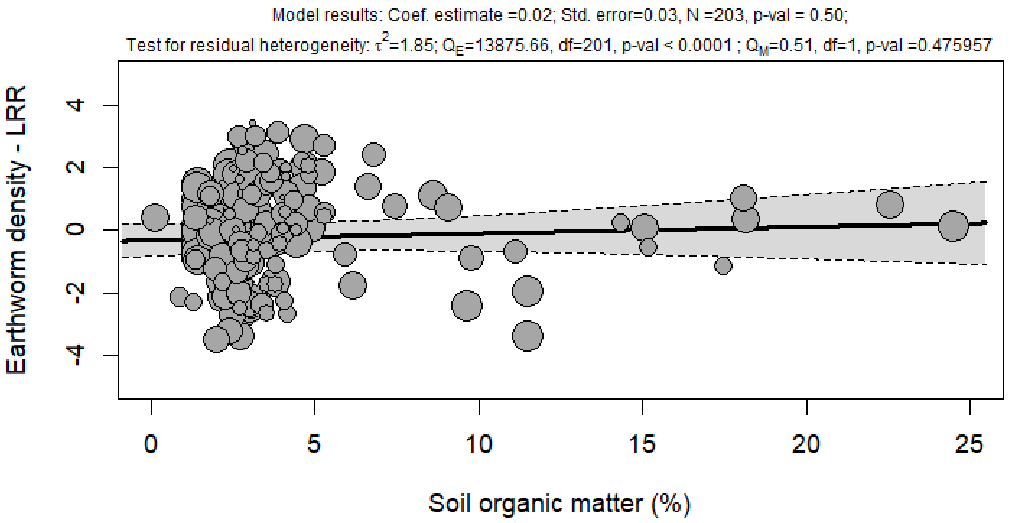

b)

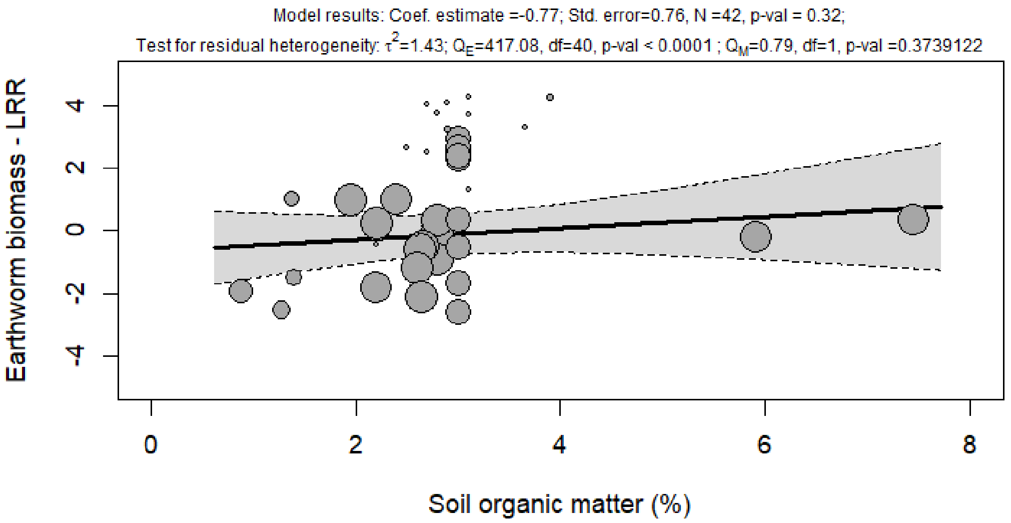

c)

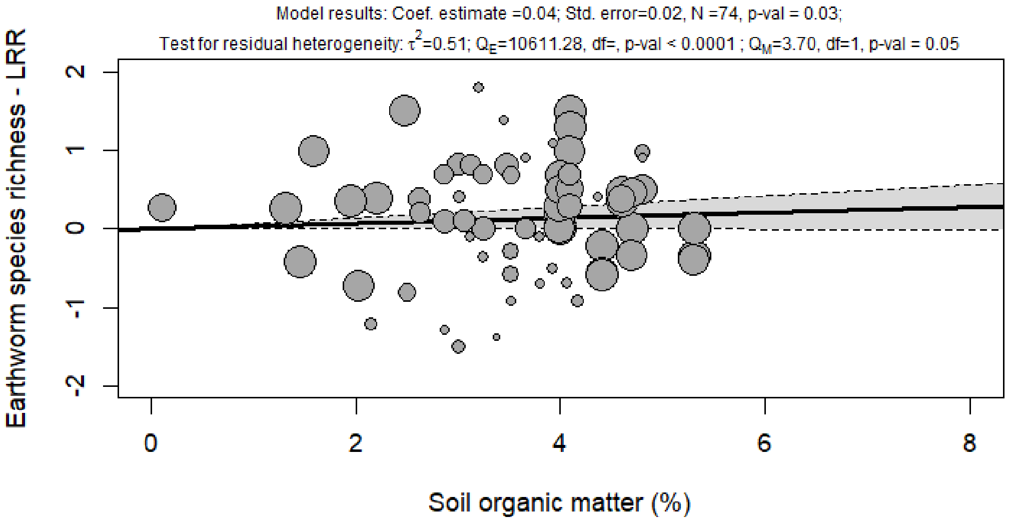
Model results: Coef. estimate =0.04; Std. error=0.02, N =74, p-val = 0.03;
Test for residual heterogeneity: τ^2=0.51; Q_E=10611.28, df=, p-val < 0.0001 ; Q_M=3.70, df=1, p-val = 0.05
Earthworm species richness - LRR
2
1
0
-1
-2
0
2
4
6
8
Soil organic matter (%)

Soil bulk density

Effect of intensified land use on earthworm a) density, b) biomass and c) species richness in relation to the soil bulk density documented in the studies. The size of a regression bubble indicates the weight of each observation in the model and displays the mean effects. Gray, dotted lines indicate the bounds of the confidence interval over the regression line.

a)

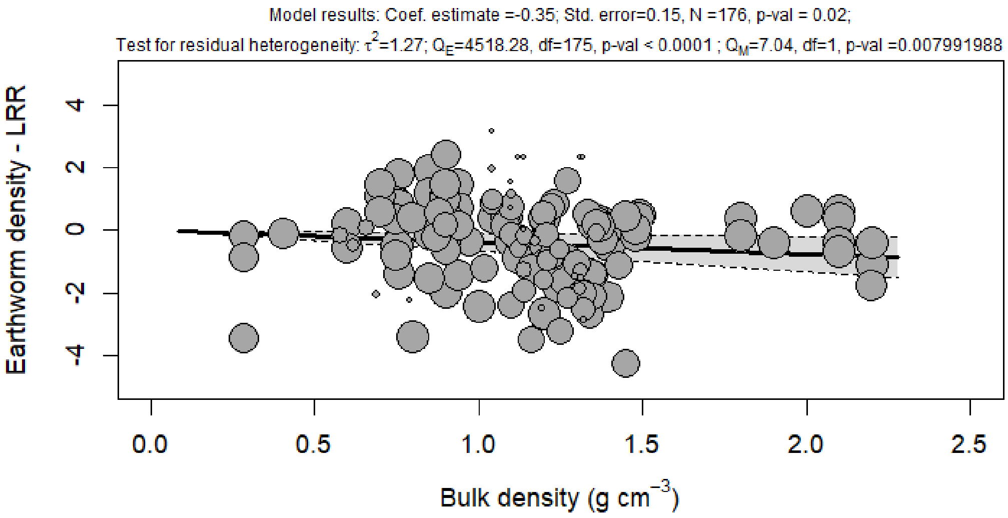

b)

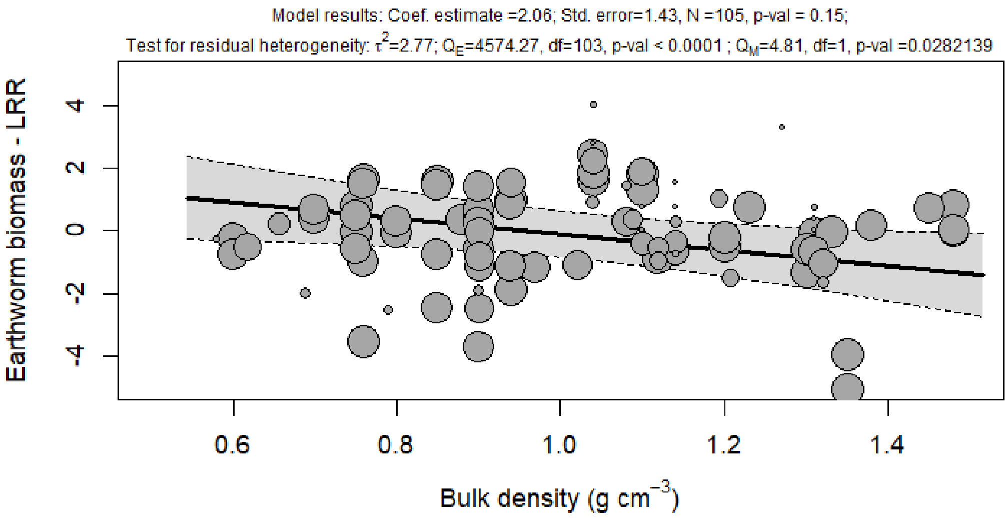

c)

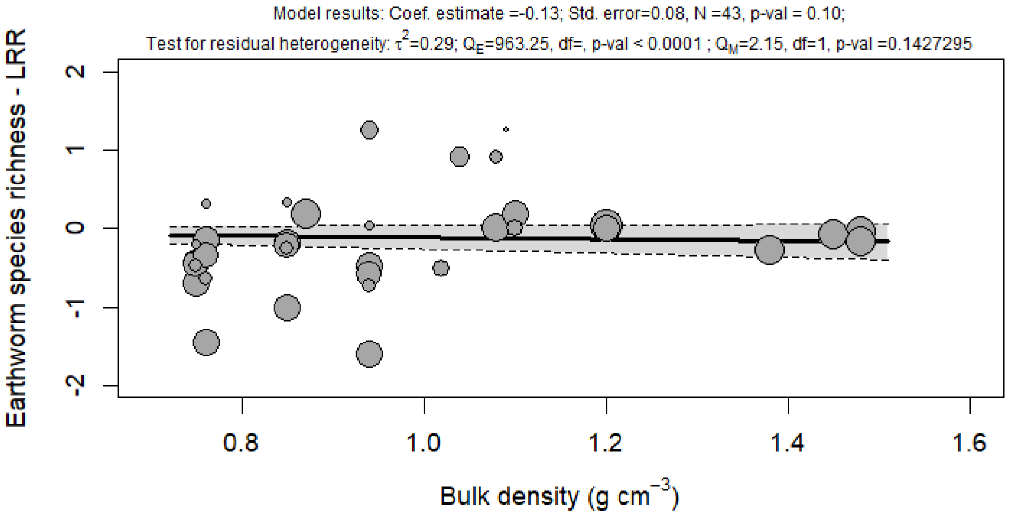
Model results: Coef. estimate =-0.13; Std. error=0.08, N =43, p-val = 0.10;
Test for residual heterogeneity: τ2=0.29; QE=963.25, df=, p-val < 0.0001 ; QM=2.15, df=1, p-val =0.1427295
Earthworm species richness - LRR
2
1
0
-1
-2
0.8
1.0
1.2
1.4
1.6
Bulk density (g cm−3)

Earthworm extraction method

Effect of intensified land use on earthworm a) density, b) biomass and c) species richness, for the various earthworm extraction methods documented in the studies. Forest plot illustrates the average effect, with the 95% confidence intervals presented in brackets, and the sample sizes for each group enclosed in parentheses.

a)

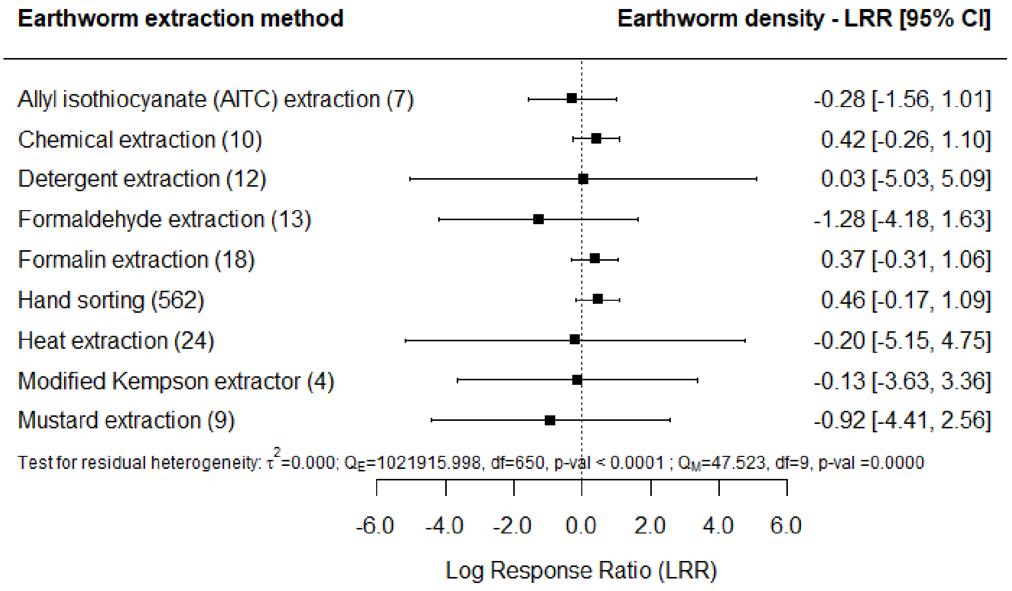

b)

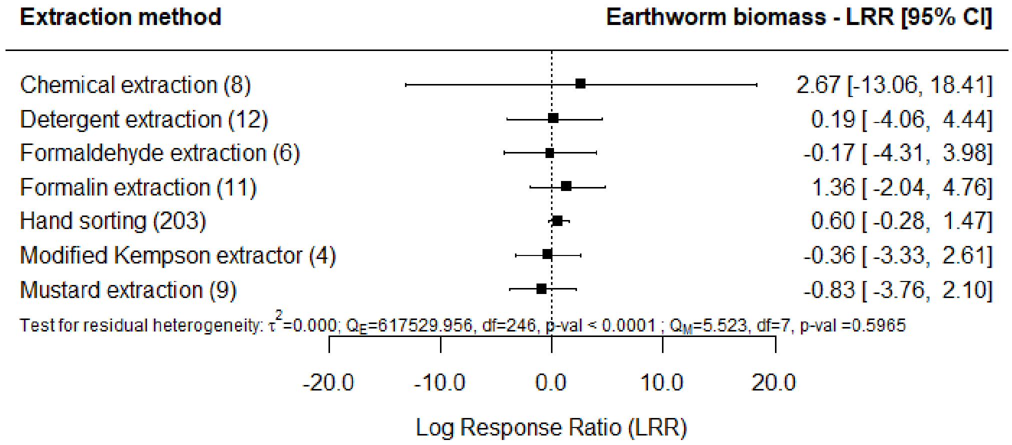
Extraction method
Earthworm biomass - LRR [95% CI]
Chemical extraction (8)
Detergent extraction (12)
Formaldehyde extraction (6)
Formalin extraction (11)
Hand sorting (203)
Modified Kempson extractor (4)
Mustard extraction (9)
2.67 [-13.06, 18.41]
0.19 [-4.06, 4.44]
-0.17 [-4.31, 3.98]
1.36 [-2.04, 4.76]
0.60 [-0.28, 1.47]
-0.36 [-3.33, 2.61]
-0.83 [-3.76, 2.10]
Test for residual heterogeneity: τ2=0.000; QE=617529.956, df=246, p-val < 0.0001 ; QM=5.523, df=7, p-val =0.5965
-20.0
-10.0
0.0
10.0
20.0
Log Response Ratio (LRR)

c)

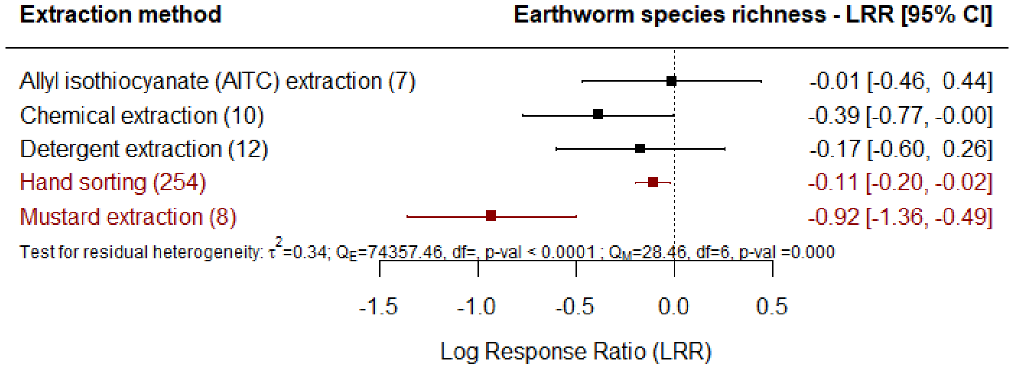
Extraction method
Earthworm species richness - LRR [95% CI]
Allyl isothiocyanate (AITC) extraction (7)
Chemical extraction (10)
Detergent extraction (12)
Hand sorting (254)
Mustard extraction (8)
-0.01 [-0.46, 0.44]
-0.39 [-0.77, -0.00]
-0.17 [-0.60, 0.26]
-0.11 [-0.20, -0.02]
-0.92 [-1.36, -0.49]
Test for residual heterogeneity: τ2=0.34; QE=74357.46, df=, p-val < 0.0001 ; QM=28.46, df=6, p-val =0.000
-1.5
-1.0
-0.5
0.0
0.5
Log Response Ratio (LRR)

Estimated effects of management and climatic zone separately for each land use

Effect of intensified land use on earthworm a) density, b) biomass and c) species richness, for the various land uses and management practices documented in the studies. N refers to the number of pairwise comparisons on which the estimation of the effect size was conducted.

a) Effects on earthworm density

Studied factor	Intensified land-use	Level of the studied factor	Effect size	Standard error	p-value	N
Fertilization	Arable cropland	Mineral + Organic	-3,14	3,81	0,411	21
Fertilization		Not fertilized	0,72	2,29	0,754	18
Fertilization		Organic	1,32	2,36	0,578	22
Fertilization	Managed grassland	Not fertilized	0,93	2,69	0,729	6
Fertilization		Organic	2,16	2,81	0,442	9
Fertilization	Managed plantation	Mineral + Organic	-4,01	4,59	0,383	12
Fertilization		Not fertilized	-0,11	2,90	0,970	3
Fertilization		Organic	0,56	3,51	0,874	3
Fertilization	Orchard	Organic	2,62	3,97	0,510	7
Fertilization	Pasture	Organic	0,22	2,99	0,942	4
Pesticide application	Arable cropland	Herbicide + Insecticide	0,26	2,44	0,915	10
Pesticide application		Insecticide	1,44	18,52	0,938	9
Pesticide application		None	-8,84	1,97	<0,0001	28
Pesticide application		Unspecified pesticide	-0,08	1,80	0,964	12
Pesticide application	Managed grassland	None	-9,53	2,27	<0,0001	7
Climatic zone	Arable cropland	Am	0,98	1,09	0,374	18
Climatic zone		Aw	1,50	0,73	0,051	20
Climatic zone		BSh	0,66	1,05	0,532	10
Climatic zone		Bwk	2,50	0,94	0,010	5
Climatic zone		Cfa	2,70	9,20	0,769	88
Climatic zone		Cfb	1,17	0,82	0,163	78

Studied factor	Intensified land-use	Level of the studied factor	Effect size	Standard error	p-value	N
Climatic zone		Csa	2,37	0,76	0,004	3
Climatic zone		Cwa	1,28	1,24	0,318	32
Climatic zone		Cwb	-2,88	0,73	0,001	27
Climatic zone		Dfa	1,88	0,86	0,038	3
Climatic zone		Dfb	1,64	1,05	0,129	6
Climatic zone	Crop livestock integration	Am	-1,57	2,89	0,587	6
Climatic zone	Fallow	Aw	0,17	0,27	0,525	7
Climatic zone	Fallow	Cwa	-2,68	1,66	0,115	12
Climatic zone	Field margins	Cfb	-0,73	1,11	0,515	3
Climatic zone		Csa	0,58	1,57	0,711	3
Climatic zone	Managed forest	Am	0,72	0,69	0,300	10
Climatic zone		Cwa	-1,96	2,97	0,516	12
Climatic zone		Cwb	0,11	0,41	0,782	4
Climatic zone	Managed grassland	Cfb	-0,14	1,22	0,909	36
Climatic zone	Managed plantation	Am	0,23	0,66	0,724	10
Climatic zone		Aw	0,45	0,50	0,373	15
Climatic zone		Cfa	1,12	9,52	0,907	7
Climatic zone	Orchard	Cfa	0,97	9,52	0,919	6
Climatic zone		Cfb	0,04	0,92	0,966	8
Climatic zone	Pasture	Am	-1,00	0,57	0,078	21
Climatic zone		Aw	-0,29	0,26	0,269	4

Studied factor	Intensified land-use	Level of the studied factor	Effect size	Standard error	p-value	N
Climatic zone		Cfa	1,42	9,22	0,877	18
Climatic zone		Csa	0,58	0,24	0,016	5

b) Effects on earthworm biomass

Studied factor	Intensified land-use	Level of the studied factor	Effect size	Standard error	p-value	N
Fertilization	Arable cropland	Mineral + Organic	0,34	5,99	0,955	7
Fertilization		Not fertilized	-8,84	3,51	0,998	4
Fertilization		Organic	-1,79	7,98	0,823	13
Climatic zone		Am	-7,44	12,80	0,562	6
Climatic zone		Aw	1,23	1,34	0,368	13
Climatic zone		Bwk	-3,87	1,59	0,019	5
Climatic zone		Cfa	1,31	1,49	0,386	6
Climatic zone		Cfb	1,01	1,49	0,502	20
Climatic zone		Cwa	-5,71	2,06	0,008	7
Climatic zone		Cwb	-3,51	1,34	0,016	9
Climatic zone		Dfa	2,56	1,40	0,081	3
Climatic zone		Dfb	-0,12	8,86	0,990	3
Climatic zone	Fallow	Aw	-0,90	0,53	0,097	5
Climatic zone		Cwa	-10,50	1,45	<0,0001	12
Climatic zone	Managed forest	Cwa	-8,19	2,09	<0,0001	12
Climatic zone		Cwb	-3,85	1,16	0,001	4
Climatic zone	Managed grassland	Cfb	0,07	2,06	0,972	16
Climatic zone	Managed plantation	Aw	-1,16	0,58	0,051	7
Climatic zone	Pasture	Am	-7,62	12,07	0,528	5

c) Effects on earthworm diversity

Studied factor	Intensified land-use	Level of the studied factor	Effect size	Standard error	p-value	N
Fertilization	Arable cropland	Mineral + Organic	0,70	0,60	0,248	5
Fertilization		Organic	-0,42	0,55	0,442	9
Fertilization	Managed grassland	Not fertilized	0,36	0,77	0,639	4
Fertilization	Managed plantation	Mineral + Organic	-1,07	0,68	0,121	12
Fertilization		Organic	-0,74	0,69	0,287	4
Climatic zone	Arable cropland	Am	-1,47	0,69	0,035	12
Climatic zone		Aw	0,61	0,61	0,320	7
Climatic zone		Cfa	-0,86	0,50	0,086	46
Climatic zone		Cfb	-0,73	0,49	0,137	16
Climatic zone		Csa	0,78	0,43	0,072	5
Climatic zone		Dfa	0,62	0,61	0,309	6
Climatic zone	Fallow	Aw	-0,03	0,44	0,951	12
Climatic zone	Field margins	Cfb	-0,61	0,46	0,185	5
Climatic zone	Managed grassland	Cfb	-0,86	0,53	0,103	17
Climatic zone	Managed plantation	Aw	0,26	0,41	0,518	17
Climatic zone		Cfa	-2,07	0,42	<0,0001	4
Climatic zone	Pasture	Am	-1,11	0,59	0,062	10

Supplement 5: Meta-analysis diagnostics

Earthworm density

The analysis comprised k=691 pairwise comparisons in total. The response ratios that were observed varied between -18.8059 and 17.7513, with 50% of the estimations being negative. The true outcomes seem to be heterogeneous, based on the Q-test (Q(690)=1160172.8935, $p < 0.0001$, $\tau^2 = 6.9861$, $I^2 = 99.8519\%$). The true outcomes have a 95% prediction interval between -5.0212 and 5.3493. Therefore, even if the average result is predicted to be positive, the genuine result in certain experiments can actually be negative. Several pairwise comparisons (13 datapoints) have values bigger than ± 3.9684 and may represent possible outliers in the context of this model, according to an analysis of the studentized residuals. Several pairwise comparisons (19 datapoints) may be deemed unduly influential based on the Cook's distances. The regression test ($p = 0.6586$) did not reveal funnel plot asymmetry, while the rank correlation test did ($p < 0.0001$).

Earthworm biomass

The analysis comprised k=283 pairwise comparisons in total. The observed response ratios ranged from -14.0079 to 16.0344, and 52% of the estimations were positive. The true outcomes seem to be heterogeneous, based on the Q-test (Q(282)=606565.6053, p<0.0001, $\tau^2 = 9.8804$, $I^2 = 99.7835\%$). The true outcomes have a 95% prediction interval between -5.7368 and 6.6200. Therefore, even if the average result is predicted to be positive, the true outcomes in certain experiments can actually be negative. Seven observations with values greater than ± 3.7502 were found in the studentized residuals, which suggested that they would be possible outliers for this model. Fifteen observations might be deemed excessively important based on the Cook's distances. The rank correlation test indicated funnel plot asymmetry ($p < 0.0001$) but not the regression test ($p = 0.6510$).

Earthworm diversity

The analysis comprised k=468 pairwise comparisons in total. The response ratios that were observed varied from -3.3322 to 14.5087, with 52% of the estimations being negative. Based on the random-effects model, the estimated average response ratio was -0.1408 (95% CI: -0.2195 to -0.0621). As a result, the average outcome (z=-3.5075, p=0.0005) was substantially different from zero. The true outcomes seem to be heterogeneous based on the Q-test (Q(467)=83299.6178, p<0.0001, $\tau^2 = 0.3788$, $I^2 = 99.6910\%$), and have a 95% prediction interval between -1.3497 and 1.0680. Therefore, even though the average result is predicted to be negative, the true outcomes may actually be positive in some instances. Upon analyzing the studentized residuals, it was found that all the observations had values less than or equal to ± 3.7714. Therefore, there was no evidence of outliers within the framework of this model. Seven observations may be deemed unduly important based on Cook's distances. The regression test ($p = 0.6779$) did not reveal funnel plot asymmetry, but the rank correlation test did ($p < 0.0001$).

Supplement 6. Sensitivity analyses

Within-study correlations

Sensitivity analysis of the assumed constant correlations between effect size estimates from each study (Density: $n = 990$ observations; Biomass: $n = 312$ observations). The log response ratio are the aggregated estimates of the correlated and hierarchical effects model, using the different correlation (r) values used for the imputation of a block-diagonal covariance matrix using the R package clubSandwich[92]. The log response ratio is the pooled estimate of 20 imputed datasets. The Aikaike information criteria (AIC) was used for the selection of the correlation value. For diversity outcomes, we used only the diagonal elements from 'vi' argument as the sampling variances, hence, no correlation was assumed for this outcome.

Outcome	Assumed correlation	Log response ratio	Standard error	$p - value$
Density	r = 0.1	0.256	0.269	0.341
Density	r = 0.3	0.318	0.282	0.260
Density	r = 0.5	0.403	0.296	0.174
Density	r = 0.7	0.513	0.296	0.083
Density	r = 0.9	0.734	0.320	0.022
Biomass	r = 0.1	0.379	0.342	0.268
Biomass	r = 0.3	0.416	0.354	0.241
Biomass	r = 0.5	0.468	0.357	0.191
Biomass	r = 0.7	0.558	0.357	0.119
Biomass	r = 0.9	0.816	0.355	0.022

Random-effects hierarchical structure

Sensitivity analysis of the assumed hierarchical correlated structure of the random effects of the model (Density: $n = 990$ observations; Biomass: $n = 312$ observations). Constant correlation assumed: r = 0.1. The log response ratio are the aggregated estimates of the correlated and hierarchical effects model, using the different the different hierarchical structures for the random effects, with the R package clubSandwich[92]. For diversity outcomes, we used only the diagonal elements from 'vi' argument as the sampling variances, hence, no hierarchical structure was assumed for this outcome. The Aikaike information criteria (AIC) was used for the selection of the hierarchical structure for the model.

Outcome	Random effects structure	Log response ratio	Standard error
Density	Observation within study	0.063	0.239
Density	Study level	0.256	0.951
Density	Observation level	2.041	0.617
Biomass	Observation within study	0.289	0.350

Outcome	Random effects structure	Log response ratio	Standard error
Biomass	Study level	0.379	0.342
Biomass	Observation level	3.431	1.655

Multivariate imputation

Sensitivity of the missing-at-random assumption

Sensitivity analysis to the missing-at-random assumption of the earthworm density data using multiple imputation with delta adjustment[93].

```
 iter imp variable
  1   1  dat.vi
  1   2  dat.vi
  1   3  dat.vi
  1   4  dat.vi
  1   5  dat.vi
  2   1  dat.vi
  2   2  dat.vi
  2   3  dat.vi
  2   4  dat.vi
  2   5  dat.vi
  3   1  dat.vi
  3   2  dat.vi
  3   3  dat.vi
  3   4  dat.vi
  3   5  dat.vi
  4   1  dat.vi
  4   2  dat.vi
  4   3  dat.vi
  4   4  dat.vi
  4   5  dat.vi
  5   1  dat.vi
  5   2  dat.vi
  5   3  dat.vi
  5   4  dat.vi
  5   5  dat.vi
```

Imputation	δ	Log response ratio	Standard error	Conf. Int. (lower)	Conf. Int. (upper)	τ^2
1	0	0.016	0.145	-0.269	0.301	14.622
	2	-0.016	0.151	-0.311	0.279	15.106
	5	-0.013	0.148	-0.302	0.277	14.847
	10	0.013	0.146	-0.272	0.299	14.626
	50	-0.006	0.146	-0.292	0.279	14.601
	100	0.026	0.142	-0.253	0.306	13.107
2	0	-0.003	0.147	-0.291	0.286	13.554
	2	0.000	0.145	-0.283	0.284	13.315
	5	0.024	0.143	-0.255	0.304	13.111
	10	0.006	0.143	-0.274	0.286	13.094
	50	0.043	0.139	-0.230	0.315	11.367
	100	0.017	0.143	-0.264	0.298	11.743
3	0	0.020	0.141	-0.257	0.296	11.542
	2	0.041	0.139	-0.232	0.314	11.373
	5	0.025	0.140	-0.249	0.298	11.367
	10	0.066	0.138	-0.204	0.335	9.814
	50	0.046	0.141	-0.230	0.321	10.075
	100	0.048	0.139	-0.225	0.320	9.935
4	0	0.064	0.138	-0.206	0.334	9.820
	2	0.052	0.138	-0.218	0.322	9.827
	5	0.124	0.149	-0.168	0.417	8.959
	10	0.117	0.150	-0.178	0.412	9.034
	50	0.118	0.150	-0.176	0.411	8.994
	100	0.123	0.149	-0.169	0.416	8.962
5	0	0.119	0.149	-0.174	0.412	8.972
	2	0.140	0.156	-0.166	0.445	9.220
	5	0.136	0.157	-0.171	0.443	9.264
	10	0.136	0.156	-0.170	0.442	9.241
	50	0.139	0.156	-0.166	0.445	9.222
	100	0.137	0.156	-0.169	0.443	9.229

Sensitivity analysis to the missing-at-random assumption of the earthworm biomass data using multiple imputation with delta adjustment[93].

```
 iter imp variable
  1   1  dat.vi
  1   2  dat.vi
  1   3  dat.vi
  1   4  dat.vi
  1   5  dat.vi
  2   1  dat.vi
  2   2  dat.vi
  2   3  dat.vi
  2   4  dat.vi
  2   5  dat.vi
  3   1  dat.vi
  3   2  dat.vi
  3   3  dat.vi
  3   4  dat.vi
  3   5  dat.vi
  4   1  dat.vi
  4   2  dat.vi
  4   3  dat.vi
  4   4  dat.vi
  4   5  dat.vi
  5   1  dat.vi
  5   2  dat.vi
  5   3  dat.vi
  5   4  dat.vi
  5   5  dat.vi
```

Imputation	δ	Log response ratio	Standard error	Conf. Int. (lower)	Conf. Int. (upper)	τ^2
1	0	0.296	0.211	-0.117	0.709	12.758
	2	0.330	0.211	-0.084	0.744	12.860
	5	0.319	0.211	-0.095	0.733	12.880
	10	0.318	0.211	-0.096	0.732	12.877
	50	0.312	0.211	-0.102	0.726	12.879
	100	0.316	0.210	-0.096	0.729	11.800
2	0	0.346	0.211	-0.067	0.759	11.876
	2	0.336	0.211	-0.077	0.749	11.892
	5	0.335	0.211	-0.078	0.748	11.890
	10	0.330	0.211	-0.083	0.743	11.891
	50	0.345	0.212	-0.070	0.760	10.887
	100	0.368	0.212	-0.048	0.783	10.931
3	0	0.360	0.212	-0.056	0.776	10.942
	2	0.360	0.212	-0.056	0.776	10.941
	5	0.355	0.212	-0.060	0.771	10.941
	10	0.381	0.218	-0.045	0.808	10.251
	50	0.397	0.218	-0.030	0.823	10.268
	100	0.391	0.218	-0.035	0.818	10.274
4	0	0.391	0.218	-0.035	0.818	10.273
	2	0.388	0.218	-0.038	0.815	10.272
	5	0.474	0.250	-0.016	0.963	10.516
	10	0.476	0.250	-0.014	0.966	10.516
	50	0.475	0.250	-0.014	0.965	10.517
	100	0.475	0.250	-0.014	0.965	10.517
5	0	0.475	0.250	-0.015	0.964	10.516
	2	0.501	0.264	-0.017	1.018	10.990
	5	0.501	0.264	-0.016	1.019	10.990
	10	0.501	0.264	-0.016	1.019	10.990
	50	0.501	0.264	-0.016	1.019	10.990
	100	0.501	0.264	-0.017	1.018	10.989

Sensitivity analysis to the missing-at-random assumption of the earthworm diversity data using multiple imputation with delta adjustment[93].

```
 iter imp variable
  1   1  dat.vi
  1   2  dat.vi
  1   3  dat.vi
  1   4  dat.vi
  1   5  dat.vi
  2   1  dat.vi
  2   2  dat.vi
  2   3  dat.vi
  2   4  dat.vi
  2   5  dat.vi
  3   1  dat.vi
  3   2  dat.vi
  3   3  dat.vi
  3   4  dat.vi
  3   5  dat.vi
  4   1  dat.vi
  4   2  dat.vi
  4   3  dat.vi
  4   4  dat.vi
  4   5  dat.vi
  5   1  dat.vi
  5   2  dat.vi
  5   3  dat.vi
  5   4  dat.vi
  5   5  dat.vi
```

Imputation	δ	Log response ratio	Standard error	Conf. Int. (lower)	Conf. Int. (upper)	τ^2
1	0	-0.114	0.058	-0.228	-0.001	1.333
	2	-0.090	0.043	-0.174	-0.007	0.633
	5	-0.224	0.032	-0.288	-0.161	0.384
	10	-0.106	0.051	-0.206	-0.007	1.116
	50	-0.248	0.033	-0.312	-0.184	0.404
	100	-0.165	0.033	-0.230	-0.100	0.168
2	0	-0.161	0.033	-0.225	-0.098	0.159
	2	-0.177	0.032	-0.239	-0.114	0.153
	5	-0.165	0.032	-0.229	-0.102	0.162
	10	-0.180	0.032	-0.243	-0.118	0.153
	50	-0.172	0.034	-0.238	-0.106	0.160
	100	-0.170	0.034	-0.236	-0.104	0.159
3	0	-0.175	0.033	-0.240	-0.109	0.158
	2	-0.172	0.033	-0.238	-0.107	0.159
	5	-0.176	0.033	-0.241	-0.110	0.158
	10	-0.174	0.034	-0.241	-0.107	0.161
	50	-0.174	0.034	-0.241	-0.107	0.161
	100	-0.175	0.034	-0.242	-0.108	0.161
4	0	-0.174	0.034	-0.241	-0.108	0.161
	2	-0.175	0.034	-0.242	-0.109	0.161
	5	-0.176	0.035	-0.245	-0.108	0.165
	10	-0.176	0.035	-0.245	-0.108	0.165
	50	-0.176	0.035	-0.245	-0.108	0.165
	100	-0.176	0.035	-0.245	-0.108	0.165
5	0	-0.177	0.035	-0.245	-0.108	0.165
	2	-0.177	0.035	-0.245	-0.108	0.165
	5	-0.177	0.035	-0.245	-0.108	0.165
	10	-0.177	0.035	-0.246	-0.108	0.165
	50	-0.177	0.035	-0.245	-0.108	0.165
	100	-0.177	0.035	-0.246	-0.108	0.165

Sensitivity of the imputation method

Sensitivity analysis of the multivariate imputation by chained equations used to estimate missing standard deviations for control and treatment observations[94]. Assumptions: Constant correlation r = 0.1; Hierarchical correlated structure of random effects. m corresponds to the number of imputed datasets over which a pooled estimate of the effect size (log response ratio) will be obtained.

Outcome	Imputation method	m	Log response ratio	Standard error
Density	Without imputation	Not applicable	0.201	0.405
**Density**	**Predictive mean matching**	**20**	**0.267**	**0.281**
Density	Predictive mean matching	50	0.279	0.287
Density	Predictive mean matching	100	0.263	0.276
Density	Classification and regression trees	20	0.221	0.266
Density	Classification and regression trees	50	0.224	0.277
Density	Classification and regression trees	100	0.224	0.276
Biomass	Without imputation	Not applicable	0.224	0.439
**Biomass**	**Predictive mean matching**	**20**	**0.388**	**0.351**
Biomass	Predictive mean matching	50	0.345	0.358
Biomass	Predictive mean matching	100	0.357	0.355
Biomass	Classification and regression trees	20	0.449	0.312
Biomass	Classification and regression trees	50	0.497	0.372
Biomass	Classification and regression trees	100	0.505	0.371
Diversity	Without imputation	Not applicable	-0.175	0.122
**Diversity**	**Predictive mean matching**	**20**	**-0.165**	**0.043**
Diversity	Predictive mean matching	50	-0.164	0.049

Outcome	Imputation method	m	Log response ratio	Standard error
Diversity	Predictive mean matching	100	-0.183	0.151
Diversity	Classification and regression trees	20	-0.168	0.043
Diversity	Classification and regression trees	50	-0.172	0.043
Diversity	Classification and regression trees	100	-0.170	0.042

Note: The imputation method selected for meta-analyses is highlighted in bold.

Supplement 7. PRISMA flow diagram. *n* refers to the number of research articles and *obs* to the number of pairwise observations

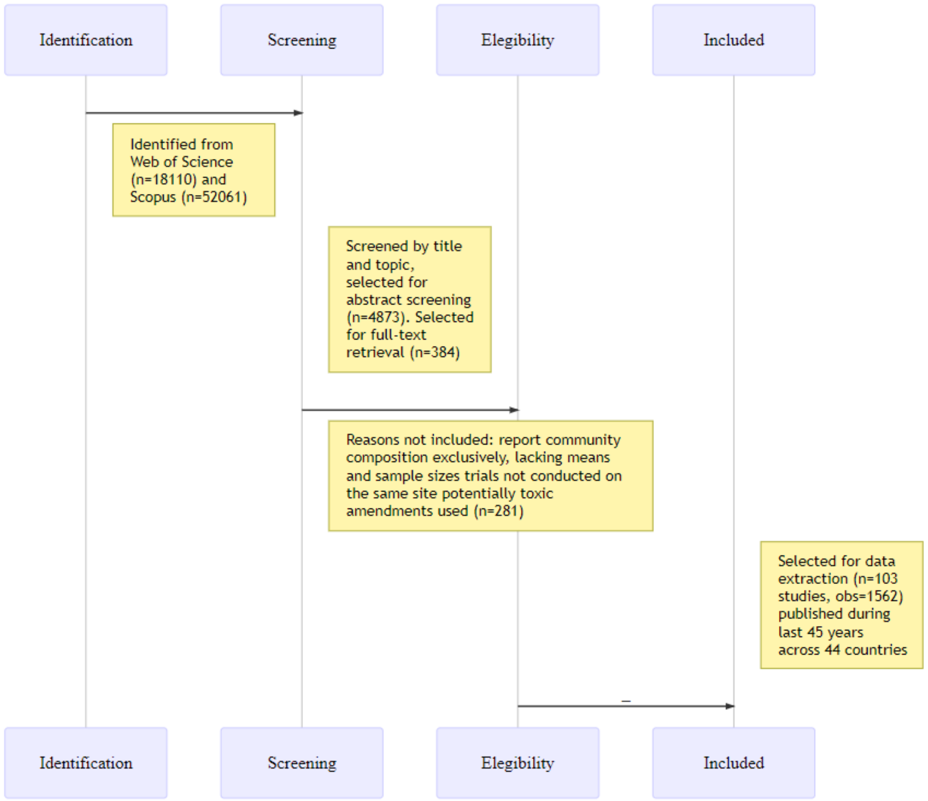

Supplement 8. Intensification effects on earthworm density per study

Reference	Estimate	Std.error	p-value	Observations
[2]	-0.4063	0.0903	<0.0001	8
[3]	1.5127	0.5885	0.0104	2
[4]	-0.1062	0.238	0.6632	16
[5]	1.62	0.2165	<0.0001	4
[95]	0.8047	0.2657	0.0026	34
[6]	1.663	0.8199	0.092	16
[7]	-1.0246	0.0729	<0.0001	3
[8]	-1.1079	0.0879	<0.0001	2
[9]	0.5547	0.7145	0.4562	36
[10]	-0.3471	0.1451	0.0171	1
[11]	0.9595	0.1379	<0.0001	8
[12]	-0.7056	0.0652	<0.0001	5
[13]	-0.1289	0.486	0.7961	4
[14]	1.7953	0.284	<0.0001	2
[15]	-0.1675	1.422	0.9063	5
[16]	0.7308	0.24	0.0249	12
[63]	12.7691	353201.1819	0.9996	4
[96]	-0.6273	0.0787	<0.0001	4
[1]	0.9808	29.8362	0.9738	1
[17]	-1.0395	0.0824	<0.0001	12
[19]	0.5208	0.8467	0.5447	12
[20]	0.7253	0.3179	0.0391	3
[21]	1.0768	6.3977	0.8664	4
[22]	-0.0333	0.2166	0.8777	3
[23]	-1.4489	0.3815	0.0004	2
[24]	-1.6135	0.4759	0.0011	2
[25]	-1.631	0.4238	0.0002	2
[97]	-3.452	0.1704	<0.0001	3
[26]	-2.467	0.3227	<0.0001	6
[27]	0.1143	0.1893	0.5608	8
[98]	0.319	0.5161	0.5368	2
[28]	-0.5202	0.2911	0.0744	8
[29]	0.3899	0.1493	0.0094	12
[30]	0.3006	0.1049	0.0043	16
[31]	-0.9274	0.0946	<0.0001	5
[32]	-0.8905	0.0882	<0.0001	10
[34]	-0.3141	0.2853	0.3083	7
[35]	1.188	0.2463	<0.0001	6
[36]	0.8505	0.2704	0.029	4

Reference	Estimate	Std.error	p-value	Observations
[37]	-0.9953	2.5122	0.6921	3
[38]	0.3198	0.1283	0.0164	4
[39]	-0.6331	0.0365	<0.0001	2
[40]	0.0518	0.5307	0.9254	12
[41]	0.054	0.0673	0.4227	12
[42]	-0.0763	0.2278	0.7379	24
[43]	-0.0128	0.1037	0.9017	8
[44]	-0.4114	0.345	0.2713	8
[45]	8.6736	0.0122	<0.0001	12
[46]	-0.9227	0.187	0.0001	5
[47]	-0.1288	0.9876	0.8966	3
[49]	2.657	0.7667	0.003	13
[50]	-1.2476	0.263	<0.0001	1
[51]	0.1586	0.4486	0.7238	4
[52]	-0.5274	0.0613	<0.0001	6
[53]	-4.2132	0.1244	<0.0001	6
[54]	-0.5674	0.1116	<0.0001	6
[55]	3.3521	3.9809	0.4001	6
[56]	-0.9899	0.6936	0.1582	2
[57]	0.1569	0.1543	0.3095	9
[99]	15.9343	0.1164	<0.0001	6
[58]	-0.0625	0.7037	0.9292	1
[59]	0.0465	0.329	0.8914	14
[60]	-0.1787	0.0734	0.0151	11
[61]	0.2919	0.7472	0.7017	4
[62]	2.9749	1.1676	0.0383	28
[66]	-2.2825	0.398	<0.0001	5
[67]	-0.2646	1.3526	0.8501	9
[68]	-0.3283	0.0295	<0.0001	3
[71]	-0.321	0.2111	0.129	3
[100]	-0.1517	0.0533	0.0046	2
[72]	-0.7616	0.1421	<0.0001	15
[73]	0.9339	0.8452	0.3027	12
[74]	-0.6112	0.2674	0.0226	25
[75]	-0.2979	0.1935	0.1242	2
[76]	1.1186	0.2193	0.0003	3
[77]	-0.3604	0.5656	0.5653	24
[78]	-0.2106	0.0611	0.0006	3
[79]	-1.0814	0.097	<0.0001	4
[80]	0.0188	0.2597	0.9424	2
[81]	1.1742	0.0536	<0.0001	5
[82]	0.3566	0.2354	0.1303	4
[83]	0.933	0.1669	<0.0001	8

Reference	Estimate	Std.error	p-value	Observations
[84]	1.2851	0.135	<0.0001	5
[86]	1.0369	0.5703	0.3768	28
[87]	-0.3075	0.033	<0.0001	4
[89]	0.293	0.1521	0.0545	5
[90]	0.2764	0.056	0.0006	14
[91]	2.8735	0.6783	0.0001	2

Supplement 9. Intensification effects on earthworm biomass per study

Reference	Estimate	Std.error	p-value	Observations
[3]	2.393	1.510.574	0.9874	2
[5]	12.381	0.3622	0.0007	4
[9]	11.673	10.143	0.2674	36
[11]	-0.7808	0.2431	0.0015	7
[12]	-0.4671	0.0625	<0.0001	5
[13]	-0.1006	11.263	0.9289	4
[15]	-0.5802	94.879	0.9513	5
[96]	-0.7773	0.0922	<0.0001	4
[19]	0.3084	79.443	0.9691	12
[20]	17.918	28.998	0.5372	3
[27]	0.9369	0.2674	0.0008	8
[28]	0.6661	0.3453	0.0549	8
[29]	0.2448	0.2088	0.2426	12
[30]	0.282	0.1384	0.0427	16
[33]	12.916	22.514	0.5669	9
[34]	-0.9676	16.623	0.5611	10
[36]	0.156	0.6045	0.7975	4
[38]	10.705	16.138	0.5078	4
[39]	-0.7134	0.0311	<0.0001	2
[40]	0.1453	0.4908	0.7689	12
[43]	0.4083	0.1133	0.0004	8
[45]	84.496	0.0131	<0.0001	12
[46]	-0.3732	124.158	0.976	2
[47]	-0.0135	117.603	0.9991	3
[101]	-0.7326	0.4307	0.0923	4
[48]	-0.7157	0.4348	0.101	2
[49]	31.032	12.883.156	0.9981	13
[50]	0.1144	0.137	0.4046	3
[51]	0.0599	0.2376	0.8013	4
[52]	-0.1549	0.087	0.0762	6
[53]	-27.149	0.1419	<0.0001	7
[57]	10.001	0.4915	0.043	9
[64]	29.746	76.889	0.6993	8
[71]	-0.103	0.2409	0.6693	2
[100]	-0.0532	0.0532	0.3182	2
[75]	-0.0402	0.2192	0.8545	2
[76]	12.361	0.5838	0.0357	3
[79]	-11.445	0.1686	<0.0001	4
[84]	22.634	0.0925	<0.0001	5

Reference	Estimate	Std.error	p-value	Observations
[87]	0.0234	0.0396	0.5555	4
[89]	-21.888	0.063	<0.0001	11
[91]	20.008	20.377	0.3272	2

Supplement 10. Intensification effects on earthworm species richness per study

Reference	Estimate	Std.error	p-value	Observations
[95]	0.1113	0.187	0.5521	34
[6]	0.2832	0.1116	0.0118	24
[13]	0.07	0.3384	0.8365	8
[16]	-0.6804	0.1353	<0.0001	12
[19]	-0.5355	0.137	0.0001	24
[21]	-0.113	0.5848	0.8481	4
[22]	-0.8999	0.3137	0.0045	3
[98]	0.0598	0.2417	0.8046	4
[28]	0.2877	0.6258	0.6461	1
[30]	-0.4708	0.108	<0.0001	20
[31]	-0.3822	0.2122	0.0728	5
[32]	-0.382	0.1516	0.0123	10
[33]	-1.1947	0.2492	<0.0001	9
[34]	0.074	0.2133	0.7289	6
[36]	0.4273	0.3368	0.2069	4
[38]	1.0858	0.2732	0.0001	4
[39]	-1.2285	0.3987	0.0023	2
[40]	-0.1647	0.1787	0.3574	12
[41]	-1.106	0.1853	0.0001	9
[43]	-0.131	0.135	0.3326	12
[45]	9.565	5274.1667	0.9986	4
[50]	0.3748	0.3276	0.2536	2
[54]	-0.0505	0.196	0.7968	6
[59]	-0.105	0.0905	0.2473	28
[62]	0.9928	0.1889	<0.0001	7
[65]	-0.0717	0.3289	0.8288	6
[71]	-0.1127	0.2814	0.6891	3
[75]	-0.3848	0.3619	0.2886	2
[78]	0.0792	2.7868	0.9773	2
[79]	-0.9249	0.1681	<0.0001	8
[82]	0.4688	0.2682	0.0816	4
[83]	0.3847	0.1323	0.0039	16
[84]	0.2592	0.1995	0.1951	6
[85]	-0.3736	0.1934	0.0544	7

References

1. Albuquerque, M. P. de, Machado, A. M. B., Machado, A. de F., Victoria, F. de C. & Morselli, T. B. G. A. Fauna edafica em sistema de plantio homogêneo, sistema agroflorestal e em mata nativa em dois municípios do rio grande do sul, brasil. *Biociências* **17**, (2009).
2. Andriuzzi, W. S., Pulleman, M. M., Cluzeau, D. & Peres, G. Comparison of two widely used sampling methods in assessing earthworm community responses to agricultural intensification. *Applied Soil Ecology* **119**, 145–151 (2017).
3. Araujo, Y. & Lopez-Hernandez, D. Earthworm populations in a savanna-agroforestry system of venezuelan amazonia. *Biology and Fertility of Soils* **29**, 413–418 (1999).
4. Ayuke, F. O. *et al.* Agricultural management affects earthworm and termite diversity across humid to semi-arid tropical zones. *Agriculture, ecosystems & environment* **140**, 148–154 (2011).
5. Barros, E. *et al.* Development of the soil macrofauna community under silvopastoral and agrosilvicultural systems in amazonia. *Pedobiologia* **47**, 273–280 (2003).
6. Bartz, M. L. C. *et al.* Earthworm richness in land-use systems in santa catarina, brazil. *Applied Soil Ecology* **83**, 59–70 (2014).
7. Bedano, J. C., Dominguez, A., Arolfo, R. & Wall, L. G. Effect of good agricultural practices under no-till on litter and soil invertebrates in areas with different soil types. *Soil & Tillage Research* **158**, 100–109 (2016).
8. Bedano, J. C., Dominguez, A., Arolfo, R. & Wall, L. G. Effect of good agricultural practices under no-till on litter and soil invertebrates in areas with different soil types. *Soil and Tillage Research* **158**, 100–109 (2016).
9. Bhadauria, T. *et al.* Earthworm populations in a traditional village landscape in central himalaya, india. *Applied Soil Ecology* **53**, 83–93 (2012).
10. Boag, B., Palmer, L., Neilson, R., Legg, R. & Chambers, S. Distribution, prevalence and intensity of earthworm populations in arable land and grassland in scotland. *Annals of Applied Biology* **130**, 153–165 (1997).
11. Brown, G. G. *et al.* No-tillage greatly increases earthworm populations in parana state, brazil. *Pedobiologia* **47**, 764–771 (2003).
12. Cameron, A. *et al.* Distribution of earthworm communities in agroecosystems with forested riparian buffer strips: A multiscale study. *Applied Soil Ecology* **167**, 104035 (2021).
13. Carnovale, D., Baker, G., Bissett, A. & Thrall, P. Earthworm composition, diversity and biomass under three land use systems in south-eastern australia. *Applied Soil Ecology* **88**, 32–40 (2015).
14. Cluzeau, D. *et al.* Integration of biodiversity in soil quality monitoring: Baselines for microbial and soil fauna parameters for different land-use types. *European Journal of Soil Biology* **49**, 63–72 (2012).
15. Cornwell, E. Effects of different agricultural systems on soil quality in northern limon province, costa rica. *REVISTA DE BIOLOGIA TROPICAL* **62**, 887–897 (2014).

16. Darmawan, A., Atmowidi, T., Manalu, W. & Suryobroto, B. Land-use change on mount gede, indonesia, reduced native earthworm populations and diversity. *Australian Journal of Zoology* **65**, 217–225 (2017).

17. De Valença, A. W. *et al.* Land use as a driver of soil fertility and biodiversity across an agricultural landscape in the central peruvian andes. *Ecological Applications* **27**, 1138–1154 (2017).

18. Decaens, T., Lavelle, P. M., Jimenez Jaen, J. J., Escobar, G. & Rippstein, G. Impact of land management on soil macrofauna in the oriental llanos of colombia. *European Journal of Soil Biology* (1994).

19. Decaens, T. & Jimenez, J. J. Earthworm communities under an agricultural intensification gradient in colombia. *Plant and Soil* **240**, 133–143 (2002).

20. Decaens, T., Bureau, F. & Margerie, P. Earthworm communities in a wet agricultural landscape of the seine valley (upper normandy, france): The 7th international symposium on earthworm ecology · cardiff · wales · 2002. *Pedobiologia* **47**, 479–489 (2003).

21. Demetrio, W. *et al.* Earthworm species in various land use systems in the campos gerais region of lapa, parana, brazil. *Zootaxa* **4496**, 503–516 (2018).

22. Demetrio, W. *et al.* Comparison of soil invertebrate communities in organic and conventional production systems in southern brazil. (2020).

23. Dominguez, A., Bedano, J. C. & Becker, A. R. Cambios en la comunidad de lombrices de tierra (annelida: Lumbricina) como consecuencia del uso de la tecnica de siembra directa en el centro-sur de cordoba, argentina. *Ciencia del suelo* **27**, 11–19 (2009).

24. Dominguez, A., Bedano, J. C. & Becker, A. R. Negative effects of no-till on soil macrofauna and litter decomposition in argentina as compared with natural grasslands. *Soil & Tillage Research* **110**, 51–59 (2010).

25. Dominguez, A., Bedano, J. C. & Becker, A. R. Negative effects of no-till on soil macrofauna and litter decomposition in argentina as compared with natural grasslands. *Soil and Tillage Research* **110**, 51–59 (2010).

26. Dominguez, A. & Bedano, J. C. Earthworm and enchytraeid co-occurrence pattern in organic and conventional farming: Consequences for ecosystem engineering. *Soil Science* **181**, 148–156 (2016).

27. Emmerling, C. Long-term effects of inundation dynamics and agricultural land-use on the distribution of soil macrofauna in fluvisols. *Biology and fertility of soils* **20**, 130–136 (1995).

28. Fang, P. *et al.* Assessing bioindication with earthworms in an intensively farmed rural landscape (yuanqiao and daqiao villages in qianjiang municipality, located in hubei province, subtropical china). *Critical reviews in plant sciences* **18**, 429–455 (1999).

29. Feijoo-Martinez, A., Zuñiga, M. C., Quintero, H., Carvajal-Vanegas, A. F. & Ortiz, D. P. Patrones de asociacion entre variables del suelo y usos del terreno en la cuenca del rio la vieja, colombia. *Acta zoologica mexicana* **26**, 151–164 (2010).

30. Feijoo, A., Carvajal, A. F., Zuñiga, M. C., Quintero, H. & Fragoso, C. Diversity and abundance of earthworms in land use systems in central-western colombia. *Pedobiologia* **54**, S69–S75 (2011).

31. Felten, D. & Emmerling, C. Effects of bioenergy crop cultivation on earthworm communities-a comparative study of perennial (miscanthus) and annual crops with consideration of graded land-use intensity. *Applied Soil Ecology* **49**, 167–177 (2011).

32. Felten, D. & Emmerling, C. Effects of bioenergy crop cultivation on earthworm communities-a comparative study of perennial (miscanthus) and annual crops with consideration of graded land-use intensity. *Applied Soil Ecology* **49**, 167–177 (2011).

33. Fragoso, C. *et al.* Agricultural intensification, soil biodiversity and agroecosystem function in the tropics: The role of earthworms. *Applied soil ecology* **6**, 17–35 (1997).

34. Fragoso, C. *et al.* Earthworm communities of tropical agroecosystems: Origin, structure and influence of management practices. *Earthworm management in tropical agroecosystems* 27–55 (1999).

35. Franco, A. L. *et al.* Loss of soil (macro) fauna due to the expansion of brazilian sugarcane acreage. *Science of the Total Environment* **563**, 160–168 (2016).

36. Fraser, P., Haynes, R. & Williams, P. Effects of pasture improvement and intensive cultivation on microbial biomass, enzyme activities, and composition and size of earthworm populations. *Biology and fertility of soils* **17**, 185–190 (1994).

37. Ge, B. *et al.* Lower land use intensity promoted soil macrofaunal biodiversity on a reclaimed coast after land use conversion. *Agriculture, Ecosystems & Environment* **306**, 107208 (2021).

38. Geissen, V., Pena-Pena, K. & Huerta, E. Effects of different land use on soil chemical properties, decomposition rate and earthworm communities in tropical mexico. *Pedobiologia* **53**, 75–86 (2009).

39. Gonzalez, G., Zou, X. & Borges, S. Earthworm abundance and species composition in abandoned tropical croplands: Comparisons of tree plantations and secondary forests. *Pedobiologia* **40**, 385–391 (1996).

40. Gormsen, D. *et al.* Management of plant communities on set-aside land and its effects on earthworm communities. *European journal of soil biology* **40**, 123–128 (2004).

41. Grosso, E., Jorge, G. & Brown, G. G. Exotic and native earthworms in various land use systems of central, southern and eastern uruaguay. *Embrapa Florestas-Artigo em periodico indexado (ALICE)* (2006).

42. Guariento, E. *et al.* Management intensification of hay meadows and fruit orchards alters soil macro-invertebrate communities differently. *Agronomy* **10**, 767 (2020).

43. Guei, A. M. & Tondoh, J. E. Ecological preferences of earthworms for land-use types in semi-deciduous forest areas, ivory coast. *Ecological Indicators* **18**, 644–651 (2012).

44. Haynes, R. & Tregurtha, R. Effects of increasing periods under intensive arable vegetable production on biological, chemical and physical indices of soil quality. *Biology and Fertility of Soils* **28**, 259–266 (1999).

45. Haynes, R., Dominy, C. & Graham, M. Effect of agricultural land use on soil organic matter status and the composition of earthworm communities in KwaZulu-natal, south africa. *Agriculture, Ecosystems & Environment* **95**, 453–464 (2003).

46. Hendrix, P., Mueller, B., Bruce, R., Langdale, G. & Parmelee, R. Abundance and distribution of earthworms in relation to landscape factors on the georgia piedmont, USA. *Soil Biology and Biochemistry* **24**, 1357–1361 (1992).

47. Hernandez, E., Perez, Y. del C., Jimenez-Garcia, D., Patron, J. C. & Bernal, H. Management and health of three corn farming systems in the region of llanos de san juan, puebla, mexico. *Agroecology and Sustainable Food Systems* **41**, 76–97 (2017).

48. Høfer, H. *et al.* Structure and function of soil fauna communities in amazonian anthropogenic and natural ecosystems. *European Journal of Soil Biology* **37**, 229–235 (2001).

49. Jordan, D., Miles, R. J., Hubbard, V. C. & Lorenz, T. Effect of management practices and cropping systems on earthworm abundance and microbial activity in sanborn field: A 115-year-old agricultural field. *Pedobiologia* **48**, 99–110 (2004).

50. Joshi, N., Dabral, M. & Maikhuri, R. K. Density, biomass and species richness of earthworms in agroecosystems of garhwal himalaya, india. *Tropical Natural History* **10**, 171–179 (2010).

51. Juarez-Ramon, D. & Fragoso, C. Comunidades de lombrices de tierra en sistemas agroforestales intercalados, en dos regiones del centro de mexico. *Acta zoologica mexicana* **30**, 637–654 (2014).

52. Kamdem, P. V. A. N., Michel And Otomo. Distribution and diversity of earthworm (annelida, clitellata) populations across four land use types in northern cameroon. *Turkish Journal of Zoology* **42**, 79–89 (2018).

53. Kanianska, R., Jaďuďova, J., Makovnikova, J. & Kizekova, M. Assessment of relationships between earthworms and soil abiotic and biotic factors as a tool in sustainable agriculture. *Sustainability* **8**, 906 (2016).

54. Kernecker, M., Whalen, J. K. & Bradley, R. L. Agricultural management and flooding shape habitats for non-native earthworms in southern quebec, canada. *Applied Soil Ecology* **96**, 240–250 (2015).

55. Kraft, E. *et al.* Edaphic fauna affects soybean productivity under no-till system. *Scientia Agricola* **78**, (2020).

56. Lamande, M., Hallaire, V., Curmi, P., Peres, G. & Cluzeau, D. Changes of pore morphology, infiltration and earthworm community in a loamy soil under different agricultural managements. *Catena* **54**, 637–649 (2003).

57. Lavelle, P. & Pashanasi, B. Soil macrofauna and land management in peruvian amazonia (yurimaguas, loreto). *Pedobiologia (Jena)* **33**, 283–291 (1989).

58. Majeed, M. Z. *et al.* Differential impact of different land-use types on the population density and community assemblages of edaphic macroinvertebrates in district sargodha, punjab, pakistan. *Pakistan Journal of Zoology* **50**, (2018).

59. Moreno, G. *et al.* Exploring the causes of high biodiversity of iberian dehesas: The importance of wood pastures and marginal habitats. *Agroforestry Systems* **90**, 87–105 (2016).

60. Mujeeb Rahman, P., Varma, R. & Sileshi, G. Abundance and diversity of soil invertebrates in annual crops, agroforestry and forest ecosystems in the nilgiri biosphere reserve of western ghats, india. *Agroforestry systems* **85**, 165–177 (2012).

61. Nanganoa, L. T. *et al.* Impact of different land-use systems on soil physicochemical properties and macrofauna abundance in the humid tropics of cameroon. *Applied and Environmental Soil Science* **2019**, (2019).

62. Nunes, D. H., Pasini, A., Benito, N. P. & Brown, G. G. Earthworm diversity in four land use systems in the region of jaguapitã, parana state, brazil. (2006).

63. Oliveira, E. P. de. Influência de diferentes sistemas de cultivos na densidade populacional de invertebrados terrestred em solo de varzea da amazônia central. *Amazoniana: Limnologia et Oecologia Regionalis Systematis Fluminis Amazonas* **12**, 495–508 (1993).

64. Owen, R. B. & Galbraith, W. J. Earthworm biomass in relation to forest types, soil, and land use: Implications for woodcock management. *Wildlife Society Bulletin (1973-2006)* **17**, 130–136 (1989).

65. Paoletti, M. G. *et al.* Soil invertebrates as bio-indicators in a natural area converted from agricultural use: The case study of vallevecchia-lugugnana in north-eastern italy. *Journal of sustainable Agriculture* **34**, 38–56 (2009).

66. Peres, G., Bellido, A., Curmi, P., Marmonier, P. & Cluzeau, D. Relationships between earthworm communities and burrow numbers under different land use systems. *Pedobiologia* **54**, 37–44 (2010).

67. Polo Benito, N., Fatima Guimarães, M. de & Pasini, A. Characterization of management systems in a typic hapludox using biological, physical and chemical parameters. *Semina Ci. agr.* 473–484 (2008).

68. Ponge, J.-F. *et al.* The impact of agricultural practices on soil biota: A regional study. *Soil Biology and Biochemistry* **67**, 271–284 (2013).

69. Postma-Blaauw, M. B., Goede, R. G. M. de, Bloem, J., Faber, J. H. & Brussaard, L. Soil biota community structure and abundance under agricultural intensification and extensification. *Ecology* **91**, 460–473 (2010).

70. Postma-Blaauw, M. B., De Goede, R. G., Bloem, J., Faber, J. H. & Brussaard, L. Agricultural intensification and de-intensification differentially affect taxonomic diversity of predatory mites, earthworms, enchytraeids, nematodes and bacteria. *Applied Soil Ecology* **57**, 39–49 (2012).

71. Prendergast-Miller, M. T. *et al.* Arable fields as potential reservoirs of biodiversity: Earthworm populations increase in new leys. *Science of the Total Environment* **789**, 147880 (2021).

72. Rajwar, N., Singh, V., Bhatt, S. & Bisht, S. S. Earthworm population dynamics in three different land use systems along an altitudinal gradient (208–2609 m asl) in kumaun himalayas, india. *Tropical Ecology* 1–7 (2022).

73. Rakotomanga, D. *et al.* Crop management and soil macrofauna diversity in the highlands of madagascar. *Biotechnologie, Agronomie, Societe et Environnement* **20**, 495–507 (2016).

74. Rossi, J.-P. & Blanchart, E. Seasonal and land-use induced variations of soil macrofauna composition in the western ghats, southern india. *Soil biology and biochemistry* **37**, 1093–1104 (2005).

75. Rudisser, J., Tasser, E., Peham, T., Meyer, E. & Tappeiner, U. Hidden engineers and service providers: Earthworms in agricultural land-use types of south tyrol, italy. *Sustainability* **13**, 312 (2020).

76. Ruiz-Cobo, D. H., Feijoo, A. & Rodriguez, C. Comunidades de marcoinvertebrados edaficos en diferentes sistemas de uso del terreno en la cuenca del rio otun, colombia. *Acta zoologica mexicana* **26**, 165–178 (2010).

77. Silva, R. F. da, Aquino, A. M. de, Mercante, F. M. & Guimarães, M. de F. Population of earthworm (annelida: Oligochaeta) in a hapludox under soil used systems. *Ciencia Rural* **36**, 673–677 (2006).

78. Singh, S., Singh, J. & Vig, A. P. Diversity and abundance of earthworms in different landuse patterns: Relation with soil properties. *Asian J Biol Life Sci* **9**, 111–118 (2020).

79. Smith, R. G. *et al.* Diversity and abundance of earthworms across an agricultural land-use intensity gradient. *Soil and Tillage Research* **100**, 83–88 (2008).

80. Steinwandter, M., Schlick-Steiner, B. C., Seeber, G. U. H., Steiner, F. M. & Seeber, J. Effects of alpine land-use changes: Soil macrofauna community revisited. *Ecology and Evolution* **7**, 5389–5399 (2017).

81. Suarez, L. R., Salazar, J. C. S., Casanoves, F. & Bieng, M. A. N. Cacao agroforestry systems improve soil fertility: Comparison of soil properties between forest, cacao agroforestry systems, and pasture in the colombian amazon. *Agriculture, Ecosystems & Environment* **314**, 107349 (2021).

82. Talavera, J. *et al.* Anthropogenic disturbance and environmental factors drive the diversity and distribution of earthworms in sao miguel island (azores, portugal). *Applied Soil Ecology* **145**, 103301 (2020).

83. Tondoh, J. E., Monin, L. M., Tiho, S. & Csuzdi, C. Can earthworms be used as bio-indicators of land-use perturbations in semi-deciduous forest? *Biology and Fertility of Soils* **43**, 585–592 (2007).

84. Tondoh, J. E., Guei, A. M., Csuzdi, C. & Okoth, P. Effect of land-use on the earthworm assemblages in semi-deciduous forests of central-west ivory coast. *Biodiversity and Conservation* **20**, 169–184 (2011).

85. Tsiafouli, M. A. *et al.* Intensive agriculture reduces soil biodiversity across europe. *Global change biology* **21**, 973–985 (2015).

86. Ventiņš, J. Earthworm (oligochaeta, lumbricidae) communities in common soil types under intensive agricultural practice in latvia. in *Proceedings of the latvian academy of sciences. Section b* vol. 65 48–56 (2011).

87. Vršič, S., Breznik, M., Pulko, B. & Rodrigo-Comino, J. Earthworm abundance changes depending on soil management practices in slovenian vineyards. *Agronomy* **11**, 1241 (2021).

88. Wong, M.-K. *et al.* Comparison of soil macro-invertebrate communities in malaysian oil palm plantations with secondary forest from the viewpoint of litter decomposition. *Forest Ecology and Management* **381**, 63–73 (2016).

89. Yeates, G., Shepherd, T. & Francis, G. Contrasting response to cropping of populations of earthworms and predacious nematodes in four soils. *Soil and Tillage Research* **48**, 255–264 (1998).

90. Zerbino, M. S. Efecto de rotaciones cultivo-pasturas en siembra directa, con pastoreo, sobre comunidades de oligochaeta. *Agrociencia (Uruguay)* **16**, 15–23 (2012).

91. Zou, X. & Gonzalez, G. Changes in earthworm density and community structure during secondary succession in abandoned tropical pastures. *Soil Biology and Biochemistry* **29**, 627–629 (1997).

92. Pustejovsky, J. E. & Tipton, E. Meta-analysis with robust variance estimation: Expanding the range of working models. *Prevention Science* **23**, 425–438 (2022).

93. Leacy, F. P., Floyd, S., Yates, T. A. & White, I. R. Analyses of sensitivity to the missing-at-random assumption using multiple imputation with delta adjustment: Application to a tuberculosis/HIV prevalence survey with incomplete HIV-status data. *American journal of epidemiology* **185**, 304–315 (2017).

94. Van Buuren, S. & Groothuis-Oudshoorn, K. Mice: Multivariate imputation by chained equations in r. *Journal of statistical software* **45**, 1–67 (2011).

95. Bartz, M. L. C., Pasini, A. & Brown, G. G. Earthworms as soil quality indicators in brazilian no-tillage systems. *Applied Soil Ecology* **69**, 39–48 (2013).

96. Aquino, A. M. de *et al.* Invertebrate soil macrofauna under different ground cover plants in the no-till system in the cerrado. *European journal of soil biology* **44**, 191–197 (2008).

97. Dominguez, A., Bedano, J. C., Becker, A. R. & Arolfo, R. V. Organic farming fosters agroecosystem functioning in argentinian temperate soils: Evidence from litter decomposition and soil fauna. *Applied Soil Ecology* **83**, 170–176 (2014).

98. Falco, L. B. *et al.* Earthworm assemblages in different intensity of agricultural uses and their relation to edaphic variables. *PeerJ* **3**, e979 (2015).

99. Lima, S. S. de, Aquino, A. M. de, Leite, L. F. C., Velasquez, E. & Lavelle, P. Relação entre macrofauna edafica e atributos quimicos do solo em diferentes agroecossistemas. *Pesquisa Agropecuaria Brasileira* **45**, 322–331 (2010).

100. Rajkhowa, D., Bhattacharyya, P., Sarma, A. & Mahanta, K. Diversity and distribution of earthworms in different soil habitats of assam, north-east india, an indo-burma biodiversity hotspot. *Proceedings of the national academy of sciences, India section B: biological sciences* **85**, 389–396 (2015).

101. Heyer, W., Hulsbergen, K.-J., Wittmann, C., Papaja, S. & Christen, O. Field related organisms as possible indicators for evaluation of land use intensity. *Agriculture, ecosystems & environment* **98**, 453–461 (2003).
